# Local Electric Field Controls Fluorescence Quantum Yield of Red and Far-Red Fluorescent Proteins

**DOI:** 10.3389/fmolb.2021.633217

**Published:** 2021-02-03

**Authors:** Mikhail Drobizhev, Rosana S. Molina, Patrik R. Callis, J. Nathan Scott, Gerard G. Lambert, Anya Salih, Nathan C. Shaner, Thomas E. Hughes

**Affiliations:** ^1^ Department of Cell Biology and Neuroscience, Montana State University, Bozeman, MT, United States; ^2^ Department of Chemistry and Biochemistry, Montana State University, Bozeman, MT, United States; ^3^ JNScott Consulting, LLC, Boulder, CO, United States; ^4^ Department of Neurosciences, UC San Diego, San Diego, CA, United States; ^5^ Antares & Fluoresci Research, Dangar Island, NSW, Australia

**Keywords:** red fluorescent proteins, quantum yield, two-photon absorption, twisted intramolecular charge transfer, local electric field, energy gap law, Marcus equation, molecular dynamics simulations

## Abstract

Genetically encoded probes with red-shifted absorption and fluorescence are highly desirable for imaging applications because they can report from deeper tissue layers with lower background and because they provide additional colors for multicolor imaging. Unfortunately, red and especially far-red fluorescent proteins have very low quantum yields, which undermines their other advantages. Elucidating the mechanism of nonradiative relaxation in red fluorescent proteins (RFPs) could help developing ones with higher quantum yields. Here we consider two possible mechanisms of fast nonradiative relaxation of electronic excitation in RFPs. The first, known as the energy gap law, predicts a steep exponential drop of fluorescence quantum yield with a systematic red shift of fluorescence frequency. In this case the relaxation of excitation occurs in the chromophore without any significant changes of its geometry. The second mechanism is related to a twisted intramolecular charge transfer in the excited state, followed by an ultrafast internal conversion. The chromophore twisting can strongly depend on the local electric field because the field can affect the activation energy. We present a spectroscopic method of evaluating local electric fields experienced by the chromophore in the protein environment. The method is based on linear and two-photon absorption spectroscopy, as well as on quantum-mechanically calculated parameters of the isolated chromophore. Using this method, which is substantiated by our molecular dynamics simulations, we obtain the components of electric field in the chromophore plane for seven different RFPs with the same chromophore structure. We find that in five of these RFPs, the nonradiative relaxation rate increases with the strength of the field along the chromophore axis directed from the center of imidazolinone ring to the center of phenolate ring. Furthermore, this rate depends on the corresponding electrostatic energy change (calculated from the known fields and charge displacements), in quantitative agreement with the Marcus theory of charge transfer. This result supports the dominant role of the twisted intramolecular charge transfer mechanism over the energy gap law for most of the studied RFPs. It provides important guidelines of how to shift the absorption wavelength of an RFP to the red, while keeping its brightness reasonably high.

## Introduction

**FIGURE 1:**
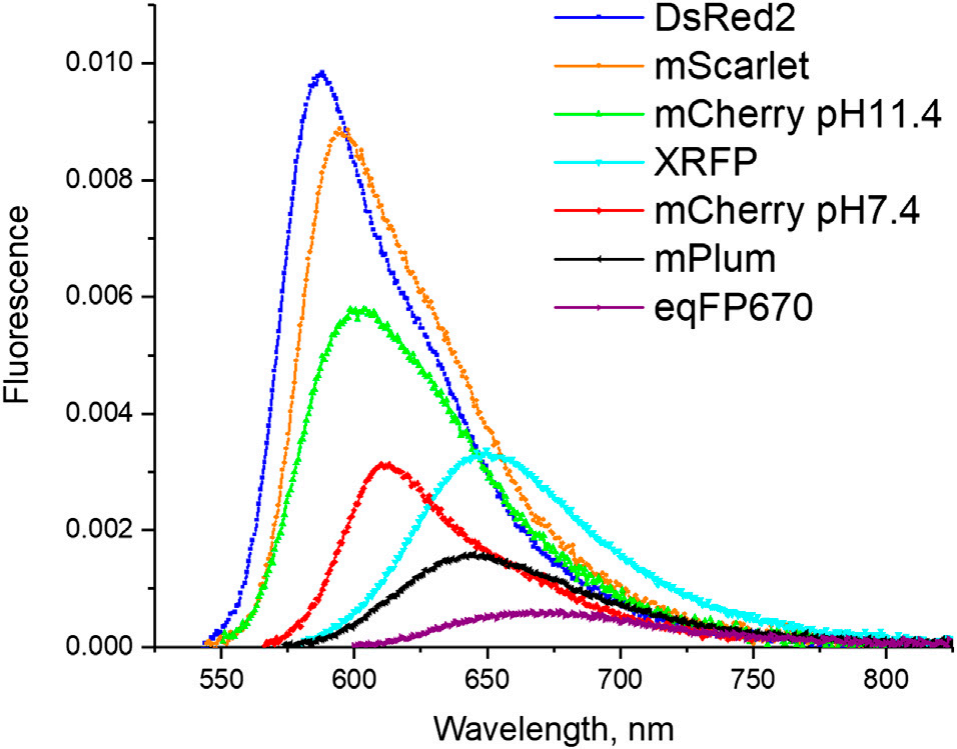
Fluorescence spectra of a set of RFPs studied in this work. The amplitude of the fluorescence intensity is normalized such that the integral under the curve is proportional to the fluorescence quantum yield.

Red fluorescent proteins (RFPs) and biosensors derived from them present an important addition to a rich palette of genetically encoded fluorescent probes widely employed in bioimaging (Tsien et al., 1998; Shaner et al., 2005; Day and Davidson, 2009; Wiedenmann et al., 2009; Chudakov et al., 2010). Their red-shifted absorption and fluorescence make it possible to report from deeper layers of tissues with less background autofluorescence compared to green fluorescent proteins (GFPs). The spectral red shifts in RFPs are due to a longer π-conjugated system in the chromophore structure, that includes an additional *N*-acylimine group appended to a common GFP chromophore, *p*-hydroxybenzylidine-imidazolinone. Although the fluorescence quantum yield (QY) of the first discovered tetrameric red FP, DsRed, was quite large (∼0.7), the most popular red-shifted monomeric mutant variants carrying the same chromophore, mPlum and mCherry, show much lower QYs: 0.1–0.2 (Shaner et al., 2005). RFPs that are further red-shifted, such as eqFP670 and mGrape3, are even dimmer, with quantum yields of ∼0.05 (Lin et al., 2009; Shcherbo et al., 2010); see Figure 1. A number of red genetically encoded calcium indicators (GECIs) also show low QYs (∼0.2) even in their active fluorescent state (Molina et al., 2019).

Fast nonradiative relaxation leading to a low QY in the reddest variants of RFPs can be due to different mechanisms. First, the shift of the emission frequency of a molecule to the red (due to chemical modifications or interactions with environment) often results in acceleration of the internal conversion, following the “energy gap law” (Englman and Jortner, 1970; Jung et al., 2012). This mechanism is quite general and simply reflects the dependence of vibrational states distribution and coupling between them (through Franck-Condon factors) on the energy gap between the two electronic terms. Another mechanism of fast relaxation involves twisted intramolecular charge transfer (TICT) that becomes possible due to structural flexibility of the chromophore in some FP variants. In this case, the rotation about one or both of the bridge methine bonds (i.e., phenolate, P, or imidazolinone, I) drives the molecular system to a twisted state that corresponds to a conical intersection of the excited and ground states potential energy surfaces. Once in this state, the molecule undergoes an ultrafast transition from the excited to the ground state. Twisting in the excited state occurs in concert with significant charge transfer (CT) across the bridge from one side of the chromophore to another (Olsen and Smith, 2007; Simine et al., 2018; Park and Rhee, 2016; Sun et al., 2012, Moron et al., 2019).

In the isolated anionic GFP chromophore, such TICT states are quasi-stable intermediates on the excited state potential energy surfaces for either I- or P-rotations (Martin et al., 2004; Altoe et al., 2005). Those states are close to conical intersection seams, but still are separated from the ground state surface by small gaps (Martin et al., 2004; Altoe et al., 2005). In contrast, twisting of the isolated anionic RFP chromophore around the P-bond leads directly to a conical intersection seam at twisting angles of ∼75°–90° (Olsen and Smith, 2007). Like in the GFP chromophore, this rotation is virtually barrierless and exergonic. In contrast, twisting around the I-bond has a barrier and is endergonic. Olsen and Smith suggested that, in contrast to the GFP chromophore, in the RFP chromophore the electronegativity of the acylimine (A) substituent plays a decisive role in selecting the P-pathway of the TICT process.

As for the chromophore inside a protein, several factors can impede its ultrafast relaxation along the TICT pathway (Tolbert et al., 2012; Jung et al., 2012). Steric clashes with the surrounding bulky groups is the most obvious one. These interactions are usually much more efficient for rotation around the I-bond and less so for the P-bond (Chudakov et al., 2003; Stiel et al., 2008) because of the larger moving volume in the former case. A volume-conserving hula-twist motion involving concert rotation around both exocyclic bonds was put forward to explain low quantum yields in some GFP mutants (Jung et al., 2005) and the RFP mPlum (Moron et al., 2019), although this mechanism was questioned for GFP in (Altoe et al., 2005).

The effect of electrostatic interactions of the chromophore with the protein surrounding (including hydrogen bonding) can also contribute to the dynamics of TICT. In one scenario, a stronger electric field directed from P to I would shift the phenolate π-conjugation resonance from a quinonoid to a benzenoid form. This would facilitate rotation around the P-bond because it becomes closer to single in character. This so-called electronic effect of controlling nonradiative relaxation was experimentally observed in certain GFP mutants. Variants having less hydrogen bonding of the phenolate oxygen (i.e., more quinonoid structure) generally showed an increased quantum yield and fluorescence lifetime compared to mutants with more hydrogen bonds (benzenoid structure) (Jung et al., 2005; Jung et al., 2012). Alternatively, if the charge transfer corresponding to a shift of electronic density from P to I upon P-rotation is a more important factor than the bond order, than applying the field from I to P would speed up the rotation through an electrostatic driving force. This particular mechanism was theoretically predicted for GFPs (Park and Rhee, 2016; Simine et al., 2018) and RFPs (Olsen and Smith, 2007; Sun et al., 2012; Moron et al., 2019), however, it was not experimentally confirmed yet. Thus, it is crucial to experimentally measure the protein internal field. Such measurements however present a serious challenge, especially in the case of the two-dimensional RFP chromophore, in which both components of the field *E* projected onto the molecular axes (*E*_*x*_ and *E*_*y*_) can be important.

In general, any of the above mechanisms, including vibrational relaxation described by the energy gap law and chromophore twisting (possibly sensitive to the electric field) can contribute to a fast nonradiative relaxation in RFPs (Figure 1). Our goal is to understand which of these processes is most important, if any. To this end, we need to evaluate the electric fields created by the protein environment at the chromophore site. A correlation (or the absence of one) between the nonradiative relaxation rate (*k*_nR_) and the amplitude and direction of the local electric field for a set of RFP variants can reveal the mechanism of relaxation. Is a redder shift inextricably connected to a faster nonradiative relaxation? We aim to answer this important question, and, eventually, reveal clues as to how to find RFP variants with large quantum yields.

## Materials and Methods

### Identification and Cloning of XRFP

XRFP was identified as part of a large survey of published raw mRNA-Seq data sets in the NCBI Sequence Read Archive (SRA) database. Transcriptomes were assembled using Trinity (Grabherr et al, 2011; Haas et al., 2013) and using the public Galaxy bioinformatics server (Afgan et al., 2018). Candidate FP-encoding transcripts were identified by BLAST homology searching using avGFP as the query against the assembled transcriptome database. For each FP homolog found, the coding region was identified and a synthetic gene was designed to produce the encoded polypeptide sequence using codons optimized for *Escherichia coli* expression using an in-house BioXP3200 instrument (SGI-DNA, La Jolla, CA) or ordered as a gBlock double-stranded gene fragment (Integrated DNA Technologies, San Diego, CA). Fragments encoding FPs were inserted using Gibson assembly (Gibson et al., 2009) into the vector pNCS for expression (see below). XRFP was identified along with green- and orange-emitting FPs from the animal (data not shown).

### Expression and Purification of Proteins

His-tagged RFPs were expressed in DH10B *E. coli* cells with bacterial expression plasmids encoding each protein. mScarlet, eqFP670, and mCherry were encoded on the pNCS vector, which contains a promoter for constitutive expression. DsRed2 and mPlum were encoded on pBAD; DsRed2-pBAD was a gift from Michael Davidson (Addgene plasmid # 54608; http://n2t.net/addgene:54608; RRID:Addgene_54608), and mPlum-pBAD was a gift from Michael Davidson and Roger Tsien (Addgene plasmid # 54564; http://n2t.net/addgene:54564; RRID:Addgene_54564). *Escherichia coli* expressing the RFPs were cultured in Circlegrow (MP Biomedicals) for 1–2 days at 30°. To induce expression of the pBAD plasmids, 0.1% w/v of L-arabinose was added to the media before inoculating. The fluorescent *E. coli* pellets were lysed with BugBuster (MilliporeSigma). Lysates were purified either with Ni-TED columns as per manufacturer’s protocol (Macherey–Nagel) or His60 Ni Superflow Resin (Clontech). For the latter, protein bound to the resin was washed 2–3 times with equilibration buffer (50 mM sodium phosphate, 300 mM sodium chloride, 20 mM imidazole; pH 7.4) ∼10 times the resin volume. Proteins purified via Ni-TED columns were eluted with buffer composed of 50 mM sodium phosphate, 300 mM sodium chloride, 250 mM imidazole; pH 8. For the His60 Ni Resin method, the elution buffer was 50 mM sodium phosphate, 300 mM sodium chloride, 300 mM imidazole; pH 7.4. Photophysical measurements were performed in elution buffer, except for mCherry pH 11.4, which was dialyzed into 25 mM sodium phosphate.

### Linear Absorption and Fluorescence Properties

Linear absorption spectra were measured with a Lambda 950 spectrophotometer (Perkin Elmer). To obtain the peak extinction coefficient of an anionic chromophore, we performed a stepwise alkaline titration up to complete denaturation of the protein. At each step, 10 μl of 0.1 M NaOH was added to 0.5 ml of protein solution, held in a spectroscopic cuvette with 1-cm optical path, followed by recording of absorption spectrum. To correct for different protein concentrations at each step, absorption spectrum was multiplied by a factor equal to the ratio of total volume to initial volume of solution. Usually a dependence of optical density of the native anionic chromophore peak versus optical density of the denatured protein peak (at 452 nm) shows a linear region. In this region of pH, there are only two species present: the anionic chromophore in native protein and denatured protein (in the same region the series of absorption spectra show an isosbestic point). The slope of this linear dependence is equal to the ratio of extinction coefficients of the two species. Using known extinction value of denatured protein, 44,100 M^−1^ cm^−1^ at 452 nm (Ward, 2005; Gross et al., 2000), we obtain the extinction coefficient of anionic species. In the cases where the immature green chromophore was present initially, its contribution to the final denatured protein concentration was subtracted.

Corrected fluorescence spectra and fluorescence quantum yields were measured with an integrating sphere fluorometer (Quantaurus-QY, Hamamtsu) using 1-cm quartz cuvettes. The peak OD of the samples was <0.1. The reference (buffer-only) measurements were done in the same cuvette.

Fluorescence lifetime was measured with a Digital Frequency Domain system ChronosDFD (ISS) appended to a PC1 ISS spectrofluorometer. Peak optical density of the samples and reference solutions in 1-cm cuvettes was kept below 0.1. Fluorescence was excited with a 518-nm laser diode (ISS, model 73292). The excitation was modulated with multiple harmonics in the range 10–300 MHz for eqFP670 and mPlum, and 5–150 MHz for the rest of the proteins. In this method, a fluorescence lifetime standard is used to obtain the instrumental response function in each individual measurement. For cross checking, we employed Rose Bengal (Sigma-Aldrich) in ethanol with *τ* = 0.78 ns (Fleming et al., 1977; Cramer and Spears, 1978) or methanol, *τ* = 0.55 ns, (Fleming et al., 1977; Cramer and Spears, 1978; Reed et al., 1981; Rodgers, 1981, Lakowicz et al., 1986); Rhodamine 6G in deionized water, *τ* = 4.0 ns (Reisfeld et al., 1988; Magde et al., 2002; O’Hagan et al., 2001); and Rhodamine B in deionized water, *τ* = 1.74 ns (Boens et al., 2007). For each RFP, we used two or three different standards and the results were similar (deviations <6%). Fluorescence of all the samples and standards was collected at 90° through two identical 561LP Edge Basic^TM^ filters (Semrock) to cut off all excitation light. In the case of eqFP670 and mCherry (pH7.4), we used an additional HQ 650/20 (Chroma) filter to selectively collect fluorescence from the red-shifted form. For mCherry at pH 11.4, we used an additional HQ 577/10 filter (Chroma) to selectively collect fluorescence of the blue-shifted form. All the modulation ratio and phase delay curves were fitted to model functions corresponding to a single exponential fluorescence decay. The corresponding *χ* ^2^ values were in the range from 0.53 to 1.16.

Corrected fluorescence excitation spectra were obtained with a LS-55 spectrofluorimeter (Perkin Elmer). For mCherry at pH 11.4, the registration wavelength was 575 nm, where the red-shifted form does not fluoresce. For all other proteins, the registration wavelength was selected at the red side of the fluorescence spectrum near its maximum.

### Two-photon Absorption Spectra, Cross Sections, and Polarization Ratio

Two-photon excitation spectra and cross sections were measured as described previously (Drobizhev et al., 2020). Briefly, we used an Insight DeepSee femtosecond laser (Spectra Physics) tunable in the 680–1,300 nm range coupled with a PC1 photon counting spectrofluorometer (ISS). LDS 798 in CDCl_3_:CHCl_3_ (2:1) solution was used as a spectral shape standard and Rhodamine 6G in MeOH was used as a 2PA cross section standard, with *σ*_2_ = 10 GM at 1060 nm. The laser beam was focused into the sample with a NIR achromatic lens, *f* = 45 mm (Edmund Optics). The sample solutions were held in 3-mm spectroscopic cuvettes (Starna) and fluorescence was collected from the first 0.7-mm layer of solution, to avoid laser absorption by the solvent. A 745SP filter (Semrock) was placed after the sample to cut the scattered light. For mCherry pH 11.4, an additional HQ 577/10 filter (Chroma) was used.

To evaluate the two-photon polarization ratio *Ω*, the same experimental system was used. Additionally, a quarter-wave plate (Thorlabs) was placed in front of the entrance diaphragm of the fluorometer and the Glan polarizer (ISS) was set after the sample in the detection optical path. The quarter-wave plate was mounted on a rotation stage (Thorlabs) which made it possible to rotate it around both the laser propagation direction and the vertical axis. This helped to adjust the rotation and tilt angles to make the polarization very close to circular at each laser wavelength. The ellipticity of polarization was <7% in the tuning range 900–1,300 nm. By definition, *Ω* is the ratio of 2P cross sections obtained under circular and linear polarization of excitation. Experimentally, we collect fluorescence at 90° to excitation, first using circularly polarized light and then linearly (vertically) polarized light. To get rid of the effect of fluorescence polarization, at each polarization of laser, two measurements were done: one with the detection polarizer set vertically, and another with the detection polarizer set horizontally. The two-photon polarization ratio was then calculated as follows (Wan and Johnson, 1994):

$$\text{Ω}=\frac{2F_{\text{OV}}+F_{\text{OH}}}{F_{\text{VV}}+2F_{\text{VH}}},$$ (1)

where *F* is the fluorescence signal, indexes O and V describe circular and vertical polarizations of excitation, respectively, and indexes V and H describe vertical and horizontal positions of detection polarizer. The sensitivity of the detector (PMT R928) for vertical versus horizontal polarization of fluorescence was checked by exciting the sample with horizontally polarized laser light and the ratio of the two signals (G-factor) was *G* = 1.0. Standard deviations of *Ω* were estimated from 10 measurements of *Ω* in the same conditions at a number of different wavelengths.

### Molecular Dynamics Simulations

#### MD Parameters

Force field parameters for the mCherry, mPlum, and DsRed chromophores were those published in (Dmitrienko et. al., 2006) with the exception of excluding the phenolic hydroxyl hydrogen in order to form the anionic chromophore.

The optimized potentials for liquid simulations (OPLS) amino acid atom types suggested by Dmitrienko et. al. were also used. Standard OPLS-AA atom types were used for the extended chain atoms. A variant of the mCherry chromophore residue force field was also constructed, wherein equilibrium bond lengths and angles of the force field were set to those found in the 2H5Q pdb crystal structure rather than those suggested by Dmitrienko et al. for the neutral DsRed chromophore.

For both the DsRed and mCherry/mPlum chromophores, hydrogen addition rules were added to the OPLS-AA force field in such a way as to complete typical valency requirements for the anionic chromophores.

#### Simulation Details

All MD simulations were done with Gromacs-5.0.2 (Pronk et al., 2013). Initial atomic charges were found by protonating the pdb structure of a given mFruit protein using Gromacs, embedding it in a box of transferable intermolecular potential with 3 points (TIP3P) solvent, adding sodium ions in sufficient number to balance the charge of the protein, and then subjecting the chromophore to a semi-empirical INDO/S2 (ZINDO) analysis in the presence of the electric field due to non-chromophore protein atoms, water molecules, and ions in the simulation cell. Initial charges were calculated in this way for mCherry, mPlum, and DsRed, with a separate initial charge set determined for mCherry with its Glu215 residue protonated.

Following determination of an initial set of atomic charges for each mFruit protein chromophore based on the electrostatic environment, simulations were carried out. Each protein/simulation was treated with 100 ps of equilibration under the NVT ensemble with the protein heavy atoms constrained by a large harmonic restoring force, followed by 400 ps of NPT equilibration using the Berendsen barostat and a further 500 ps using the Parrinello-Rahman barostat, also with heavy protein atoms constrained. Finally, protein constraints were removed and 100 ns of dynamics was carried out, with positions saved each picosecond for a total of 100001 frames. The simulation was split into individual coordinate files, which were used for all subsequent analysis.

For each simulation frame a ZINDO calculation was performed, which yielded excitation energy, wavelength, oscillator strength, transition dipole, and Δ***μ*** for the 59 lowest energy transitions. All atoms in the simulation cell that were not part of the quantum chromophore were used to calculate the electric field vector and electric potential at each chromophore atom, so that the electrostatic environment could be completely accounted for in the ZINDO calculation. The output of the ZINDO calculations was also used to determine the S_0_ and S_1_ state charges for each simulation frame. The S_1_–S_0_ charge differences for each simulation were averaged, and those average values were used along with the atomic coordinates for each simulation frame to calculate the shift in chromophore excitation energy due to each protein atom that was not part of the quantum chromophore as well as for each water molecule and ion. This straightforward Coulombic summation allowed for the unambiguous assignment of Stark shifts due to protein, water and ions for each simulation frame as well.

Following the first set of 100 ns simulations based on initial MD charges, S_0_ charge values for each quantum chromophore atom were plotted to assess charge convergence. The initial charges based on the solvated pdb structures were found to be quite close to those calculated for the chromophore during dynamics but were sufficiently different to warrant a second charge iteration. Average charge values for each MD chromophore atom were extracted from the above described plots, with an attempt made to identify portions of the trajectory where a given charge appeared to be approximately constant. These new MD chromophore atom charge values were used to begin a second set of 100 ns simulations of each RFP. Data for each simulation was analyzed using the ZINDO technique as described above, and chromophore charges during dynamics were found to be in excellent agreement with the static, assigned, second generation MD charges. After the 100 ns second charge iteration simulations, dynamics were continued for another 100 ps with frames collected every 2 fs. This high resolution data was analyzed in the same fashion as the coarser data sets described above.

## Results

### Role of Radiative and Nonradiative Relaxation Rates in Controlling Fluorescence Quantum Yield

We selected seven RFPs with a large variation of absorption/fluorescence peak wavelengths and fluorescence quantum yields. They include DsRed2 (Yanushevich et al., 2002), mCherry (Shaner et al., 2005) in pH 7.4 and in pH 11.4 buffers, having different electrostatic environment of chromophore (Shu et al., 2006), mPlum (Wang et al., 2004), mScarlet (Bindels et al., 2017), eqFP670 (Shcherbo et al., 2010), and XRFP (Shaner, 2018). Unless stated otherwise, all proteins were measured in a pH 7.4 buffer. With respect to the wild type DsRed (Matz et al., 1999), DsRed2 contains the R2A, K5E, K9T, V105A, I161T, and S197A mutations (Yanushevich et al., 2002). The optical properties of DsRed2 are very similar to DsRed (Drobizhev, et al., 2011) and, therefore, we will use the previously obtained data for DsRed as a reference, wherever possible. For consistency, we performed new independent measurements of the absorption and fluorescence photophysical properties of these proteins, Table 1.

**TABLE 1:** Absorption and fluorescence properties of RFPs.

Protein	λ_abs_ nm ±1	ε_max_ 10^3^ M^−1^ cm^−1^ ±10%	λ_fl_ nm±2	φ ±6%	τ ns±10%	*k*_*R*_ ns^−1^±12%	*k*_*nR*_ ns^−1^
DsRed2	558	103	587	0.67	3.35	0.198	0.099 ± 0.016
mScarlet	569	100	595	0.70	3.78	0.186	0.079 ± 0.013
mCherry pH 11.4	564	81	602	0.48	2.85	0.170	0.182 ± 0.020
mCherry pH 7.4	587	93	611	0.22	1.53	0.144	0.510 ± 0.051
mPlum	587	65^a^	645	0.147	1.09	0.135	0.780 ± 0.078
XRFP	575	82	650	0.31	1.79	0.170	0.385 ± 0.039
eqFP670	602	67	670	0.061	0.58	0.105	1.62 ± 0.16

The columns show, in order, maximum absorption wavelength, maximum extinction coefficient, maximum fluorescence wavelength, fluorescence quantum yield, fluorescence lifetime, radiative relaxation rate, and non-radiative relaxation rate. In cases where multiple forms of chromophore were present in solution (e.g. red and green immature forms) we provide the extinction coefficient for a major (red) form. ^a^Obtained in (Drobizhev et al., 2011) using the Strickler-Berg formula.

Since the extinction coefficient does not change very much in the series, the key parameter determining molecular brightness (*ε*_max_ *φ*) is the fluorescence quantum yield (*φ*). The quantum yield decreases about an order of magnitude when going from DsRed2 and mScarlet to the most red-shifted variant eqFP670, Table 1. The quantum yield and fluorescence lifetime depend on both the radiative relaxation rate (*k*_*R*_) and nonradiative relaxation rate (*k*_*nR*_):

$$\varphi =\frac{k_{R}}{k_{R}+k_{nR}},$$ (2)

$$\tau =\frac{1}{k_{R}+k_{nR}}.$$ (3)

**FIGURE 2:**
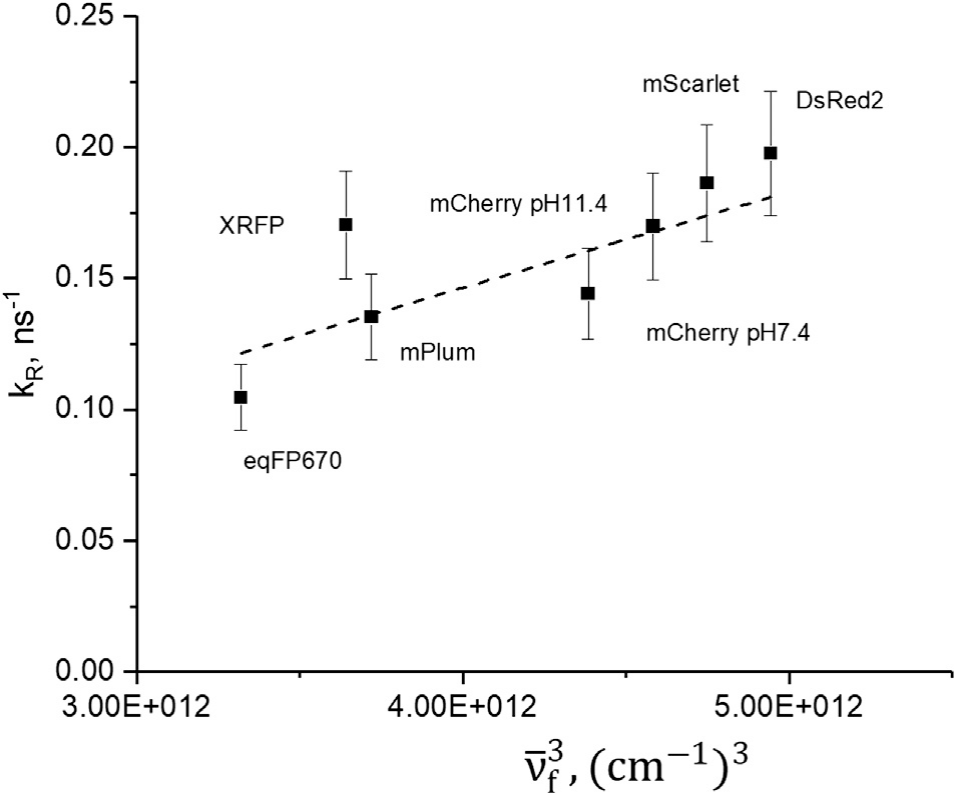
Dependence of radiative relaxation rate on cube of maximum fluorescence frequency. Dashed line represents the best fit to the Einstein equation for spontaneous emission rate.

To understand the role of *k*_*R*_ and *k*_*nR*_ in controlling quantum yield, we calculated them from the measured *φ* and *τ*,

$$k_{R}=\frac{\varphi }{\tau },$$ (4)

$$k_{nR}=\frac{1-\varphi }{\tau }\;,$$ (5)

and present them in Table 1. The dependence of *k*_R_ on the cube of fluorescence peak frequency, ${\bar{\nu }}_{f}$ (in cm^−1^), is shown in Figure 2. The dashed line is a linear fit representing the Einstein coefficient for spontaneous emission as a function of ${\bar{\nu }}_{f}^{3}$,

$$k_{R\;}=\frac{4{\left(2\pi \right)}^{4}n^{3}}{3h}\mu_{em}^{2}{\bar{\nu }}_{f}^{3}.$$ (6)

Here *n* is the refractive index of the medium and *h* is the Planck constant. The slope of the fit is proportional to the matrix element of the transition dipole moment squared *μ*_em_^2^. The slope provides an average transition dipole moment, ǀ*μ*_em_ǀ = (7.1 ± 0.2) D. In general, the *k*_*R*_ value does not change more than twofold in the series. On the other hand, *k*_*nR*_ varies about 20 times; see Table 1. We thus conclude that *k*_*nR*_ plays a decisive role in controlling quantum yield.

### Checking the Energy Gap Law

**FIGURE 3:**
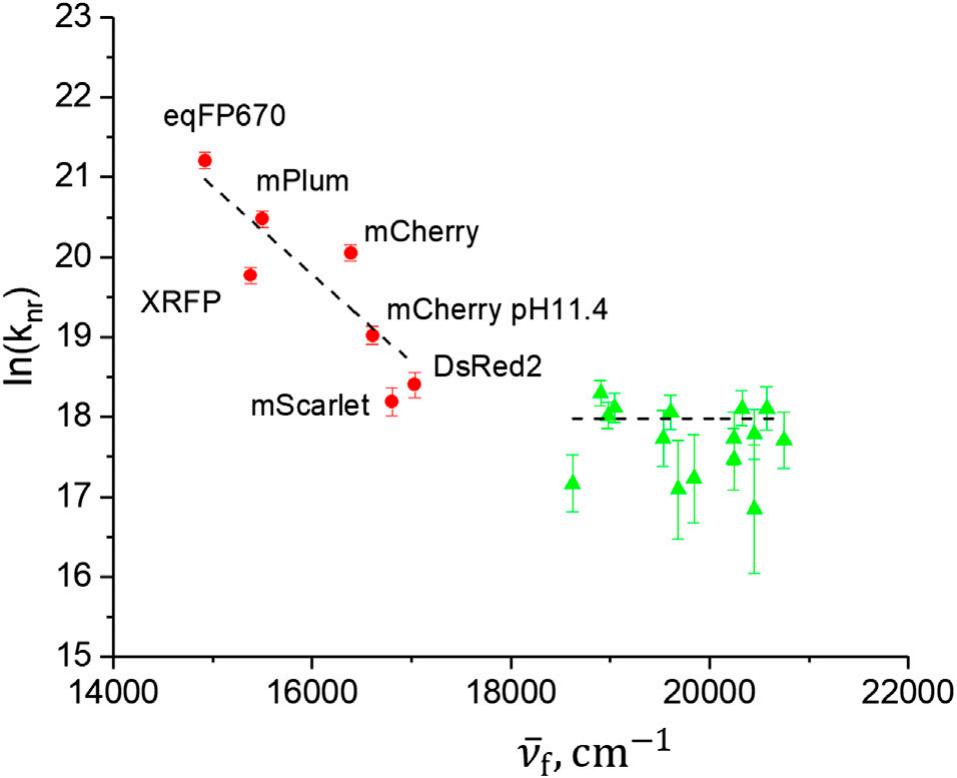
Dependence of nonradiative decay rate (log scale) on peak fluorescence frequency. Red circles correspond to RFPs studied here and green triangles correspond to a number of GFPs variants, studied previously (Drobizhev et al., 2011; Molina et al., 2017). The dashed lines represent linear fits to the two sets of data.

The energy gap law predicts an exponential decrease of *k*_*nR*_ with the increase of the energy gap Δ*E* for fluorescence transition between the energy levels of S_1_ and S_0_ states (Englman and Jortner, 1970):

$$k_{nR\;}=C\exp\left(-\frac{\xi \Delta E}{h\nu_{M}}\right),$$ (7)

where *C* is a constant factor, *ξ* is a parameter that only slowly varies with Δ*E* and *ν*_*M*_ is the maximum molecular normal vibration frequency (in Hz). Figure 3 shows the dependence of nonradiative relaxation rate (in logarithmic scale) on the fluorescence peak frequency ${\bar{\nu }}_{\text{f}}=\text{ Δ}E/\text{c}h$ for the series of RFPs studied here (red circles).

Although the RFPs qualitatively follow the predicted dependence Eq. 7, the correlation is not very strong (Pearson’s R = −0.858). In particular, although the peak fluorescence frequencies of mScarlet and mCherry pH 11.4 are very close, their *k*_*nR*_ values differ more than twofold, a difference much larger than the experimental errors. This is similarly true for the XRFP and mPlum pair. These quantitative inconsistencies suggest that the internal conversion through vibrionic coupling between S_1_ and S_0_ states, reflected in the energy gap law, is probably not the dominant mechanism of relaxation. Another, indirect support for a failure of this mechanism is obtained by considering the behavior of *k*_*nR*_ in a series of proteins with green anionic chromophore, shown in Figure 3 by green triangles. The mutants and homologues of GFP of this series were characterized previously (Drobizhev et al., 2011; Molina et al., 2017). They were selected such that they all have high quantum yields, 0.67–0.91, (to exclude other possible deactivation mechanisms, e.g., twisting around the bridge bonds), and their fluorescence spectra span a broad range, from 482 (Rosmarinus) to 537 nm (phiYFP). Due to a close similarity in the structures of the green and red chromophores, as well as closeness in shape of the absorption and fluorescence spectra in the two series, one would expect that the energy gap law, if dominant in controlling *k*_nR_, would be observed in both series. However, the “green” set does not show any correlation between *k*_*nR*_ and ${\bar{\nu }}_{fl}$. We therefore conclude that the energy gap law is not a main mechanism of relaxation in the studied set of RFPs (and GFPs) and will consider alternative mechanism in Section “*Describing the Rate of TICT Process with Marcus Formalism*”. To this end, we must first investigate the internal electric field created by the protein at the chromophore site.

### Physical Model for Determining the Protein Internal Electric Field at the Chromophore Site

The protein surrounding the chromophore creates an internal electric field that may play an important role in the mechanism of nonradiative relaxation. To determine the components of this field in the plane of the chromophore, we developed a physical model based on the following assumptions: 1) The local electric field does not change neither upon electronic excitation of the chromophore (environment is polarizable only in the ground state) nor upon twisting around exocyclic P-bond in the excited state. 2) The potentially non-homogeneous field varying from one atom to another on the chromophore will produce the same effect as a homogeneous effective field *E* ***.*** The first assumption was recently proven to a first approximation for DsRed (List et al., 2012) and a series of GFP variants (Nifosi et al., 2019). The effective field obtained after averaging the fields on several atoms of the chromophore’s π-conjugation system was also shown to describe optical properties reasonably well (Nifosi et al., 2019).

In our approach of evaluating the local electric field, we use the permanent dipole moment of the chromophore *μ* ^(*g,e*)^, where indices *g* and *e* correspond to the ground and excited states, respectively, as a linear metric of the field. In fact, if an electric field of a large magnitude (|*E*| ∼10^7^–10^8^ V/cm) is applied to a dipolar molecule with nonzero polarizability *α*, its dipole moment *μ* ^(*g,e*)^ will acquire an additional, induced, part equal to *μ* ^(*g,e*)^_ind_ = **$\underline{\alpha }$** *E* comparable to the vacuum dipole moment *μ* ^(g,e)^_0_. (The underline denotes the tensorial nature of polarizability.) Experiments and calculations show that such strong fields are indeed present in proteins (Geissinger et al., 1995; Manas et al., 1999; Schweitzer-Stenner, 2008; Callis and Burgess, 1997; Vivian and Callis, 2001). Therefore, for the linear in the field approximation, the total dipole moment reads (Atkins and Friedman, 1997):

$$\mu^{\left(g,e\right)}=\mu_{0}^{\left(g,e\right)}+\;{\underline{\alpha }}^{\left(g,e\right)}\;E.$$ (8)

For simplicity, we consider the chromophore to be planar in the ground electronic state, although small twist and tilt angles are present in some RFPs (Shu et al., 2006). We also assume here that ***μ*** ^(*e*)^ and ${\underline{\alpha }}^{\left(e\right)}$ correspond to an excited state with positions of the atomic nuclei unchanged compared to the ground state. Applying Eq. 8 separately to the ground and the excited state and then subtracting the former from the latter, we obtain:

$$\Delta \mu =\Delta \mu_{0}+\Delta \underline{\alpha }E$$ (9)

We now select *x* and *y* coordinate axes such that they correspond to the main axes of the 2x2 tensor of the polarizability change, Δ***α***, i.e., the frame where it is diagonal with components Δ*α*_xx_ and Δ*α*_yy_. In the same coordinate frame, the Δ***μ*** = ***μ*** ^(*e*)^ ***− μ*** ^(*g*)^ vector has components Δ*μ*_*x*_ and Δ*μ*_*y*_; Δ*μ*_0*,x*_ and Δ*μ*_0*,y*_ are the components of Δ***μ***_0_. Equation 9 can be projected onto the *x* and *y* coordinate axes resulting in

$$\text{Δ}\mu_{x}=\text{Δ}\mu_{0,x}+\text{Δ}\alpha_{xx}E_{x},$$ (10)

$$\text{Δ}\mu_{y}=\text{Δ}\mu_{0,y}+\text{Δ}\alpha_{yy}E_{y}.$$ (11)

(Note that in our previous papers (Drobizhev et al., 2009; Drobizhev et al., 2012a) the coefficient ½ was used in the second term of the right-hand side of Eq. 9 following an erroneous presentation in some previous literature, see e.g. (Berlin et al., 2006). A correct formula (Atkins and Friedman, 1997) does not contain it.)

If Δ*μ*_x_ and Δ*μ*_y_ were known, then it would be possible to calculate the field components *E*_x_ and *E*_y_ , by solving Eqs 10, 11:

$$E_{x}=\frac{\Delta \mu_{x}-\Delta \mu_{0,x}}{\Delta \alpha_{xx}},$$ (12)

$$E_{y}=\frac{\Delta \mu_{y}-\Delta \mu_{0,y}}{\Delta \alpha_{yy}}.$$ (13)

Now, we will demonstrate how to obtain Δ*μ*_x_ and Δ*μ*_y_ values for a chromophore within a protein, using one- and two-photon absorption spectroscopy. In our method we also rely on the quantum-mechanically calculated parameters of the isolated chromophore: transition frequency, ${\bar{\nu }}_{0}$, and components of polarizability tensor, Δ*α*_xx_ and Δ*α*_yy_, and dipole moment vector, Δ***μ***_*0,x*_ and Δ***μ***_*0,y*_ *.* This approach allows us to limit the size of the molecular system that has to be calculated quantum-mechanically to the chromophore group. We can then evaluate the parameters sensitive to the protein environment purely from experiment.

Two experimental values have to be measured in the region of the pure electronic S_0_ -> S_1_ transition: 1) the one-photon absorption frequency $\bar{\nu }$ and 2) the change of the permanent dipole moment upon excitation, Δ*μ* = |Δ***μ***|. (Here and throughout we call the pure electronic, or 0–0, transition, the one that does not involve excitation of high frequency vibrations, ${\bar{\nu }}_{v}$ > 1,000 cm^−1^). The first value can be obtained from the one-photon absorption (1PA) spectrum. The second requires the two-photon absorption (2PA) spectrum in absolute cross-section values, as well as the two-photon polarization ratio (i.e., the ratio of fluorescence signals obtained upon circular and linear two-photon excitation).

Due to the Stark effect, the chromophore experiences a spectral shift of its one-photon absorption transition frequency from ${\bar{\nu }}_{0}$ in vacuum to $\bar{\nu }$ inside the protein. Since we are dealing with strong fields such that Δ***μ*** itself depends on the field, we should include the quadratic terms in the field dependence (quadratic Stark effect). The optical transition energy for the chromophore in protein will read (Atkins and Friedman, 1997):

$$hc\bar{\nu }=hc{\bar{\nu }}_{0}-\text{Δ}\mu_{0,x}E_{x}-\text{Δ}\mu_{0,y}E_{y}-\frac{1}{2}\text{Δ}\alpha_{xx}E_{x}^{2}-\frac{1}{2}\text{Δ}\alpha_{yy}E_{y}^{2}.$$ (14)

We use the e.s.u. system of units here and throughout, unless specified otherwise. Frequency $\bar{\nu }\;$is expressed in cm^−1^, *h* is the Planck constant, and *c* is the speed of light in vacuum. Substituting Eq. 12 and Eq. 13 into Eq. 14, we obtain the relation between the transition energy and the Δ*μ*_0,x_, Δ*μ*_0,y_, Δ*μ*_x_, and Δ*μ*_y_ components:

$$hc\bar{\nu }=hc{\bar{\nu }}_{0}+\frac{\text{Δ}\mu_{0,x}^{2}}{2\text{Δ}\alpha_{xx}}+\frac{\text{Δ}\mu_{0,y}^{2}}{2\text{Δ}\alpha_{yy}}-\frac{\text{Δ}\mu_{x}^{2}}{2\text{Δ}\alpha_{xx}}-\frac{\text{Δ}\mu_{y}^{2}}{2\text{Δ}\alpha_{yy}}.$$ (15)

To find Δ*μ*_x_ and Δ*μ*_y_, it is convenient to re-group Eq. 15 as follows:

$$\frac{\text{Δ}\mu_{x}^{2}}{\text{Δ}\alpha_{xx}}+\frac{\text{Δ}\mu_{y}^{2}}{\text{Δ}\alpha_{yy}}=\frac{\text{Δ}\mu_{0,x}^{2}}{\text{Δ}\alpha_{xx}}+\frac{\text{Δ}\mu_{0,y}^{2}}{\text{Δ}\alpha_{yy}}-2hc\left(\bar{\nu }-{\bar{\nu }}_{0}\right).$$ (16)

Equation 16 represents a conic section in the (Δ*μ*_x_, Δ*μ*_y_) coordinate plane, which, depending on the signs of Δ*α*_xx_ and Δ*α*_yy_, could be either an ellipse or a hyperbola (if Δ*α*_xx_ ≠ Δ*α*_yy_). The curve is fully determined if the value of $\bar{\nu }$ is measured and Δ*μ*_*0,x*_, Δ*μ*_*0,y*_, Δ*α*_xx_, Δ*α*_yy_, and ${\bar{\nu }}_{0}$ are calculated quantum mechanically.

The second equation for finding the Δ*μ*_*x*_ and Δ*μ*_*y*_ components comes from the expression of the 2PA cross section, deduced from the second order perturbation theory in the two-level approximation of the S_0_ → S_1_ transition (Drobizhev et al., 2009) for linearly polarized degenerate excitation. The two-level approximation was justified by quantum mechanical calculations for the RFP chromophore (Drobizhev et al., 2009). The 2PA cross section corresponding to the pure electronic peak, σ_2_(0–0), depends on several factors. These are: Δ***μ*** ^2^, the extinction coefficient *ε*(0–0) (in M^−1^ cm^−1^), transition frequency $\bar{\nu }$ (we assume that the one-photon absorption peak coincides with the 0–0 transition), and *γ*, the angle between the transition dipole moment ***μ*** and the change of the permanent dipole moment Δ***μ*** :

$$\sigma_{2}\left(0-0\right)=A\frac{\text{Δ}\mu^{2}\varepsilon \left(0-0\right)\left(1+2cos^{2}\gamma \right)}{\bar{\nu }},$$ (17)

where the factor *A* is equal to

$$A=\frac{4\;10^{3}\pi \ln10f_{\text{opt}}^{2}}{5hc^{2}nN_{A}}\;.$$ (18)

Here, *N*_*A*_ is the Avogadro’s number, *f*_*opt*_ is the local field factor at optical frequency $\bar{\nu }/2$, which is usually assumed to be of a Lorentz form (Bloebmergen, 1965): *f*_*opt*_ = (*n* ^2^+2)/3.

The angle *γ* can be obtained experimentally by comparing the 2PA cross sections measured under circularly $\left(\sigma_{2}^{↺}\right)\;$ and linearly $\left(\sigma_{2}^{↕}\right)$ polarized light. Within the two-level approximation of the degenerate 2PA process, the ratio of the two measurements *Ω* depends on *γ* as follows (Meath and Power, 1984):

$$\Omega =\frac{\sigma_{2}^{↺}}{\sigma_{2}^{↕}}=\frac{\cos^{2}\gamma +3}{4\cos^{2}\gamma +2}.$$ (19)

As one can see, the polarization ratio spans the range between 2/3 and 3/2 when *γ* varies from 0° to 90°. Solving Eq. 19 for *γ* results in

$$\gamma =\pm \arccos\sqrt{\frac{2\left(1-\Omega \right)}{4\Omega -1}}+\pi n;\;n=0,1,2,\ldots$$ (20)

Solving Eq. 17 for Δ***μ*** ^2^ and noticing that ${\left|\text{Δ}\mu \right|}^{2}=\;\text{Δ}\mu_{x}^{2}+\text{Δ}\mu_{\text{y}}^{2}$, we get

$$\;\Delta \mu_{x}^{2}+\Delta \mu_{y}^{2}=\;\frac{\sigma_{2}\left(0-0\right)\bar{\nu }}{A\varepsilon \left(0-0\right)\left(1+2\cos^{2}\gamma \right)}.$$ (21)

Equation 21 presents a circle in the (Δ*μ*_x_, Δ*μ*_y_) coordinate plane with the radius equal to

$$\left|\Delta \mu \right|\;=\sqrt{\frac{\sigma_{2}\left(0-0\right)\bar{\nu }}{A\varepsilon \left(0-0\right)\left(1+2\cos^{2}\gamma \right)}}\;.$$ (22)

The curve Eq. 21 is fully determined if the values of $\bar{\nu }$, *σ*_2_(0−0), *ε*(0−0), and *γ* are measured and the constant *A* is calculated according to Eq. 18. Generally, the system of two conical sections Eq. 16 and Eq. 21 can have up to four possible solutions for the vector Δ***μ*** = (Δ*μ*_x_, Δ*μ*_y_). In Section “*Electric Fields in RFPs*” we will show how independent knowledge of the direction of the transition dipole moment in the molecular frame, as well as MD simulations can help to select the right solution.

### Parameters of the Isolated RFP Chromophore Calculated Quantum Mechanically

**FIGURE 4:**
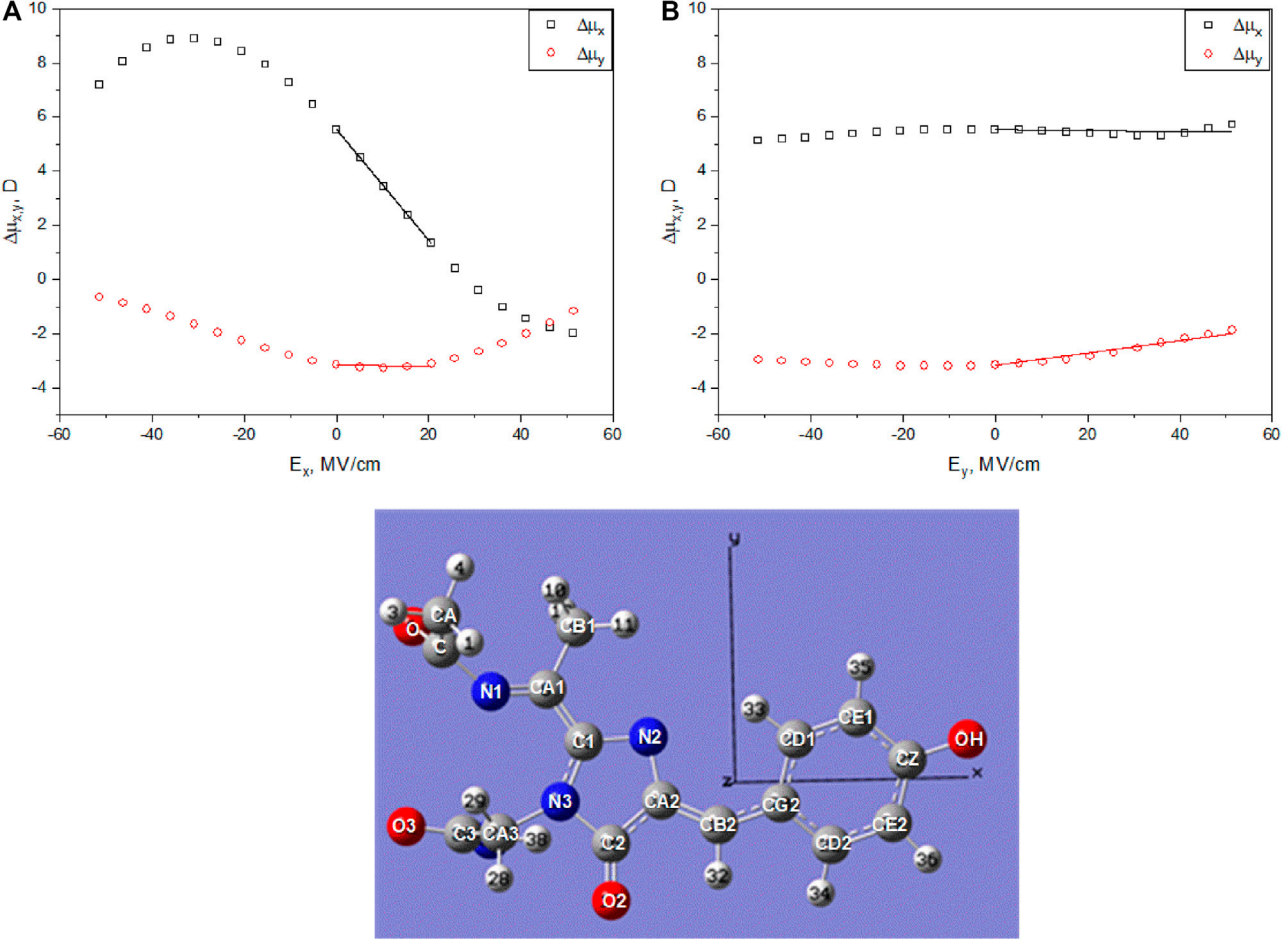
Top: Calculated permanent dipole moment differences, Δ***μ***_*x*_ (black squares) and Δ***μ***_*y*_ (red circles), between the excited and ground states of the RFP chromophore as function of applied electric field *E*_*x*_ **(A)** and *E*_*y*_ **(B)**. In the calculations, the chromophore structure was optimized for each value of the field with the phenolate and imidazolinone rings constrained to lie in the same plane (flat chromophore). Bottom: Chromophore structure optimized at zero field with the directions of axes *x* and *y* shown.

To obtain the Δ*μ*_*0,x*_, Δ*μ*_*0,y*_, Δ*α*_xx_, and Δ*α*_yy_ values, we started from the available structure of the mCherry protein (Shu et al., 2006). We cut the chromophore group (residues 65 and 66 in 2H5Q pdb file) out of the protein, added the hydrogen atoms to it, and optimized its geometry using Gaussian 09 (Frisch et al., 2016) and the B3LYP/6-311++G(d,p) density functional and basis set (Figure 4, bottom). In the optimization process, we forced the I and P rings to lie in the same plane (i.e., flat chromophore). We performed geometry optimization for a chromophore in a set of electric field values applied separately along each axis, *x* and *y*. Molecular system of coordinates was selected such that the change of polarizability tensor $\Delta \underline{\alpha }$ is diagonal in it, as described before (Drobizhev et al., 2012a), see Figure 4, bottom. Using a version of Zerner’s INDO/S2-CIS (“ZINDO”) method (Ridley and Zerner, 1973) modified to add oxygen parameters suggested by the Truhlar group (Li et al., 1999) we calculated Δ*μ*_*x*_ and Δ*μ*_*y*_ at each value of the field for the vertical optical transition. (Note that the TD-DFT often underestimates the Δ***μ*** values (Jacquemin, 2016) and ZINDO performs better than TD-DFT in calculating the RFP chromophore photophysical properties (Schaefer et al., 2007)).

Figure 4 shows the dependences of Δ*μ*_*x*_ and Δ*μ*_*y*_ on *E*_*x*_ (a) and *E*_*y*_ (b) obtained for the RFP chromophore in vacuum. We find that at zero field, Δ*μ*_*0,x*_ = 5.53D and Δ*μ*_*0,y*_ = −3.17D. The signs of the two components suggest that at least for the isolated chromophore, the electronic density shifts from P to I and also from I to A part upon excitation (by definition, the electric dipole moment is directed from negative charge to positive charge). The dependences of Δ***μ*** components on the field were fitted to linear functions (Eqs 10, 11) in the ranges of the field *E*_*x*_ = 0 ÷ 20 MV/cm and *E*_*y*_ = 0 ÷ 50 MV/cm, typical for the set of RFPs studied here (*vide infra*) with fixed zero-field values Δ*μ*_*0,x*_ and Δ*μ*_*0,y*_. The slopes of these regressions constitute the elements of the $\Delta \underline{\alpha }$ tensor: Δ*α*_xx_ = −61.3 ± 0.2 Å^3^ and Δ*α*_yy_ = 6.9 ± 0.3 Å^3^. As expected, the main component of Δ***α*** is directed along the molecular axis encountering the greater number of π-electrons, i.e., the line connecting the centers of two π-conjugated rings. The minor component is due to the A part and/or parts of conjugated system of P and I extended in a direction perpendicular to *x*.

The vacuum transition frequency ${\bar{\nu }}_{0}$ of the red FP chromophore was previously obtained in (Taguchi et al., 2009) using fragment molecular orbital (FMO) calculations and the method of configuration interaction singles with perturbative doubles, including higher-order corrections and partial renormalization, i.e., PR-CIS(Ds). This method provided an excellent agreement between the theoretical and experimental transition frequencies for the RFP chromophore within proteins. The corresponding differences between the two were 0 cm^−1^ for DsRed, −560 cm^−1^ for mCherry, and 320 cm^−1^ for mStrawberry. Transition frequencies of the isolated model chromophore with slightly different frozen conformations, corresponding to DsRed, mCherry, and mStrawberry geometry in low-temperature crystals, were calculated to be 15,890, 16,370, and 16,530 cm^−1^, respectively. For our purposes we take the average value of these three: ${\bar{\nu }}_{0}$ = 16,260 cm^−1^. We assume that it will represent well all possible conformational variations of the chromophore.

### Finding Dipole Moment Difference Δ*μ* and the Angle γ between the Vectors *μ* and Δ***μ***

**FIGURE 5:**
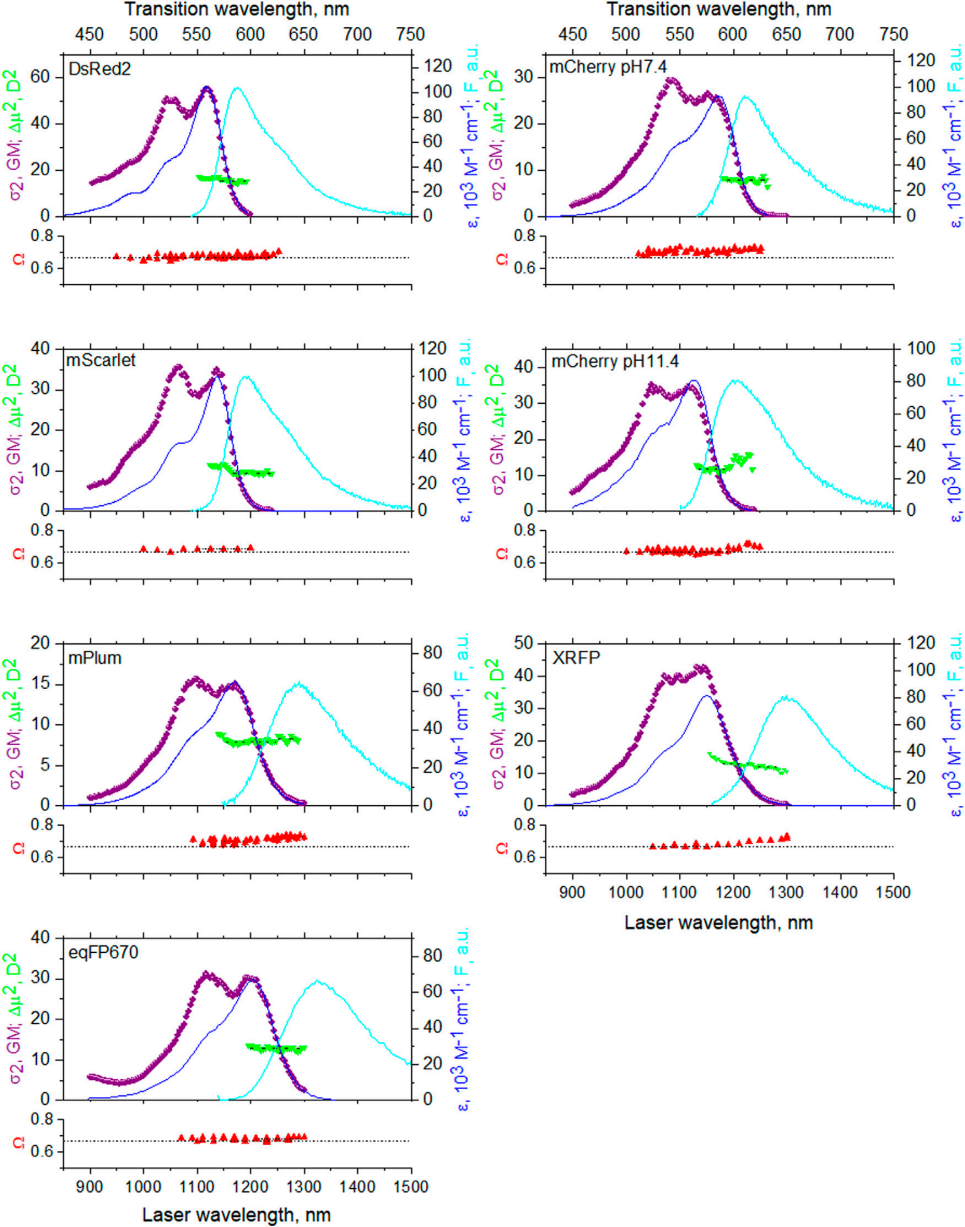
Top panels: one-photon absorption (dark blue line, right *y*-axis), fluorescence normalized to one-photon absorption peak (cyan line, right *y*-axis), and two-photon absorption (purple symbols, left *y*-axis) spectra in the region of the S_0_ -> S_1_ transition. Green triangles represent the calculated Δ***μ*** ^2^ values (left *y*-axis) as a function of excitation wavelength. Bottom panels show the two-photon polarization ratio *Ω* (red symbols) as a function of excitation wavelength. Horizontal dashed line represents a limiting case of *Ω* = 2/3, *γ* = 0. The top *x*-axis corresponds to transition wavelength, that is the same for both 1P and 2P excitation, and the bottom *x*-axis corresponds to laser wavelength, used for 2P excitation.

In this section we will find the absolute values of Δ***μ*** and the angle between ***μ*** and Δ***μ*** using one- and two-photon absorption spectroscopy. This information is necessary for evaluation of local electric fields, performed in Section “*Electric Fields in RFPs*”. The top panels of Figure 5 show one-photon absorption, fluorescence, and two-photon absorption spectra in the region of the S_0_ –>S_1_ transition of RFPs. We found that all the proteins studied here can be divided in two groups, according to their Stokes shifts values. DsRed2, mCherry, and mScarlet have smaller Stokes shifts (<1,200 cm^−1^), and mPlum, XRFP, and eqFP670 have larger Stokes shifts (≥1,500 cm^−1^). We assume that in proteins with larger Stokes shifts there are additional mechanisms of relaxation in the excited state.

It is known that the absorption spectra of mCherry at pH 7.4 and pH 11.4 (Shu et al., 2006), mPlum (Wang et al., 2004), and DsRed (Matz et al, 1999) contain contributions from at least two different forms. In particular, the mCherry spectrum always contains two contributions, one dominating at acidic pH and another at alkaline pH. In addition to a major red peak, mPlum and DsRed display a shorter wavelength minor peak, corresponding to the immature green chromophore (Gross et al., 2000; Shu et al., 2006). Therefore, in Figure 5 we present absorption spectra in the form of corrected fluorescence excitation spectra scaled to extinction coefficient of the major form. These excitation spectra were measured with the fluorescence monochromator set at a wavelength where the contributions of the minor forms were negligible. The 2PA spectra were all measured in the form of fluorescence excitation spectra and scaled to the two-photon absorption cross section. In these measurements, we again selected the excitation of the main form by choosing the appropriate observation wavelength.

At the long-wavelength side of the pure electronic transition, the shapes of the 1PA and 2PA spectra perfectly match (when plotted against transition wavelength). This demonstrates that the pure electronic transition is simultaneously allowed for both one- and two-photon absorption and its broadening does not depend on the mode of excitation. This behavior is expected for dipolar chromophores, such as the one considered here. Differences in the 2PA and 1PA spectral shapes at shorter wavelengths, i.e., in the region of vibronic transitions, can be explained by the Herzberg–Teller contribution to the Δ***μ*** factor, present only in 2PA (Drobizhev et al., 2012b). Green downward triangles in the top panels represent the wavelength dependence of |Δ***μ***|^2^ calculated according to Eqs 18, 21 in the region of pure electronic transition from independently measured σ_2_(0–0), ε(0–0), and *γ*.

The bottom panels of Figure 5 show the two-photon polarization ratio *Ω* as a function of wavelength. This value allows to calculate the angle *γ*. Analysis of the previous literature shows that the direction of transition dipole moment ***μ*** generally does not depend on the local environment in proteins with the same chromophore structure (List et al., 2012; Ansbacher et al., 2012; Nifosi et al, 2019, Myskova et al, 2020). On the other hand, the direction of Δ***μ*** is more sensitive to local electrostatics (see below). Therefore, we can consider *Ω* as a qualitative metric for the direction of Δ***μ*** within the chromophore coordinates. Furthermore, *Ω* can resolve spectrally overlapping transitions with different directions of Δ***μ***. These transitions can belong to conformers with different structures of the chromophore environment creating different local electric fields.

For the proteins with a small Stokes shift (four upper graphs in Figure 5), *Ω* is virtually constant in the region 950–1,250 nm. This points to the presence of a single electronic S_0_ –> S_1_ electronic transition in this region, cf. (Masters et al., 2018). *Ω* values between 0.67 and 0.71 correspond to small *γ* angles in these proteins, Table 2. mCherry at pH 11.4 presents an exception, where *Ω* slightly increases from 0.67 to 0.71 when going from the main peak toward the very red tail of the spectrum at λ > 1200 nm. This can be due to a minor, red-shifted, form contributing to the fluorescence signal at the long excitation wavelengths (despite selective detection of fluorescence). This is probably the form that dominates the spectrum at neutral pH with a different electrostatic environment of the chromophore (Shu et al., 2006). In fact, mCherry at pH 7.4 shows *Ω* = 0.71 across most of the spectrum and its absorption is shifted to the red, peaking near 1,200 nm (Figure 5).

**TABLE 2:** Permanent dipole moment difference vector with its length |Δ*μ*| and its direction (angle *δ* ) relatively to *x*-axis, as well as other parameters involved in finding Δ***μ***.

System	$\bar{\nu }$, cm^−1^	σ_2_(0–0), GM (±15%)	*Ω*	*γ* deg	|Δμ|, D (±5%)	*δ* (Δ***μ***) deg	*β* (*μ*) deg
Red chromophore in vacuum	*16,260* ^(*a)*^			*30÷52* ^*(b*^	6.37* *4.69* ^*(c)*^ *2.36÷3.53* ^*(b)*^	−30* *—(38÷58)* ^*(b)*^	−*(6÷9)* ^*(b)*^; −*10* ^*(d)*^
DsRed2	17,857	55	0.674±0.002	9 ± 1 *13* ^*(e)*^ *13÷16* ^*(f)*^	3.96 *7.0/f* ^*(e)*^ *3.66÷4.85* ^*(f)*^	−28 *—(17÷21)* ^*(f)*^	−19 *−(3÷6)* ^*(f)*^
mCherrypH 11.4	17731	33	0.671±0.004	7 ± 4	3.44 ± 0.05	−32	−25
mScarlet	17,575	34	0.687±0.001	15 ± 1	3.07	−38	−23
XRFP far-red XRFP red	16,821 17,483	41	0.708±0.002 0.667 ±0.002	22 ± 1 0 ± 2	3.46 3.61	−44 −35	−22 −35
mPlum far-red mPlum red	16,598 |17,094	15	0.727±0.004 0.698±0.004	26 ± 1 19	2.87 2.76 *(5±1)/f* ^*(g)*^ *2.1* ^*(h)*^	−58 −51	−32 −32
mCherry pH 7.4	17,036	24	0.714±0.002	23 ± 0.5	2.83	−51	−28 −*8.7* ^*(i)*^
eqFP670 ( IV) (III)	16,611	30	0.678±0.002	12 ± 1 —	3.58 —	−45 −135	−33 —

Column 2 shows 1PA 0-0 transition frequency, column 3 - two-photon cross section of the 0–0 transition, column 4 – two-photon polarizion ratio, (*Ω*) of the pure electronic transition, column 5 - angle *γ* between ***μ*** and Δ***μ***, column 8 − angle *β* between ***μ*** and *x*-axis. The relative random errors of measurements of σ_2_(0–0) and |Δμ| are shown in %. Literature data are presented in italic. *Calculated in this work; ^(a)^Calculated in (Taguchi et al., 2009); ^(b)^Calculated (TD-DFT CAM-B3LYP) for different conformations of red chromophore in vacuum (List et al., 2012); ^(c)^Calculated (DFT BLYP) for red chromophore in vacuum (Nifosí et al., 2007). ^(d)^Calculated in vacuum, (Ansbacher et al., 2012); ^(e)^Stark spectroscopy measurement (Lounis et al., 2001); ^(f)^Calculated (TD-DFT PE-CAM-B3LYP) for DsRed with different physical models of protein environment (List et al., 2012); ^(g)^Stark spectroscopy measurement (Abbyad et al., 2007); ^(h)^Quantum mechanical calculation (Moron et al., 2019); ^(i)^Measured in crystal using absorption of polarized light (Myskova et al., 2020). For eqFP670, both values of *α*, obtained for Δ***μ*** vector lying either in quadrant IV or III are shown.

For the proteins with a large Stokes shift (three bottom graphs in Figure 5), only eqFP670 displays a constant value of *Ω* across the spectrum. In mPlum and XRFP, *Ω* first increases and then reaches a plateau in the red tail of the 0–0 transition. Correspondingly, *γ* changes from 19° to 26° in mPlum and from 0° to 22° in XRFP. This suggests a presence of at least two different conformations of the chromophore environment in these proteins.

**FIGURE 6:**
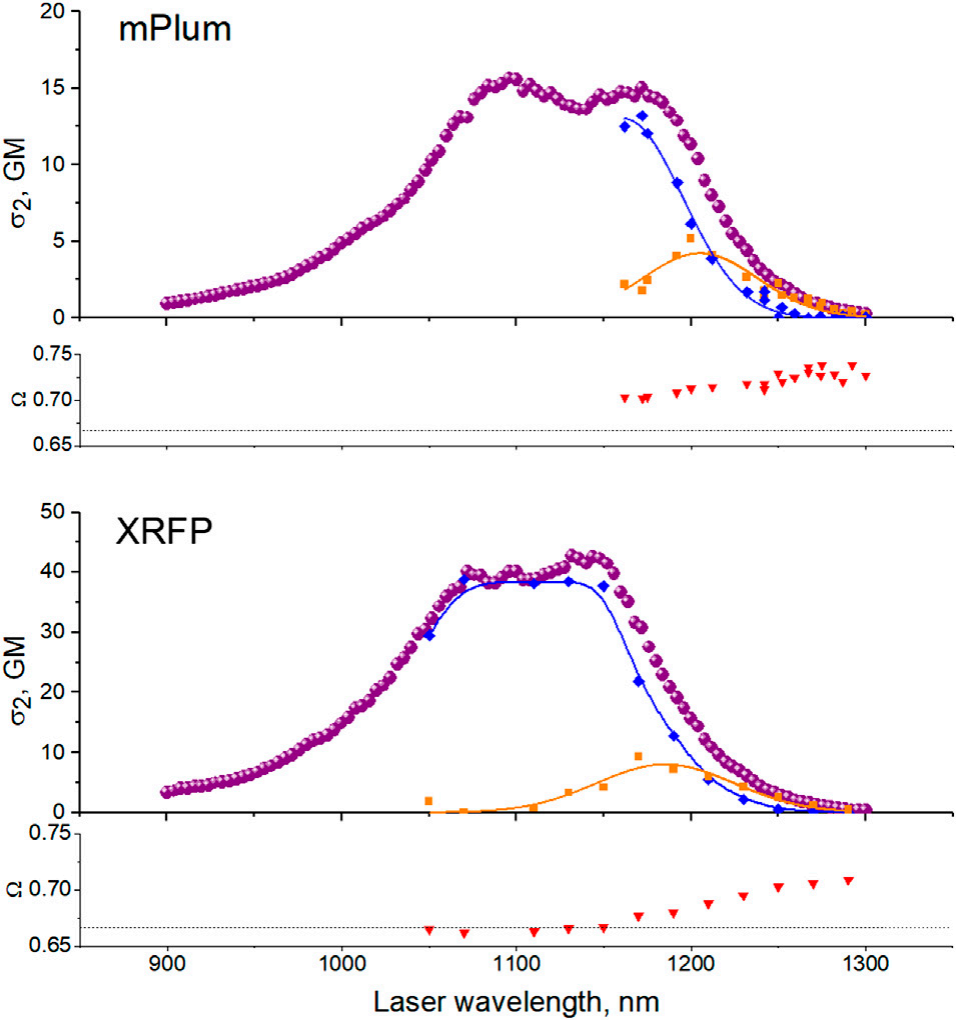
Resolution of two-photon transitions corresponding to two different conformations of the chromophore’s environment in mPlum (top) and XRFP (bottom). Top panels: Purple circles represent the total effective 2PA cross section $\sigma_{2,total}{\left(\lambda \right)}_{a}$, blue rhombs—contribution from the red from $\left(\rho_{a}\sigma_{2,a}\left(\lambda \right)\right)$ and orange squares – from the far-red form $\left(\rho_{b}\sigma_{2,b}\left(\lambda \right)\right)$. Bottom panels: Red triangles represent *Ω* values. The dashed line corresponds to the *Ω* = 2/3 (*γ* = 0) case.

It is known that in mPlum a large Stokes shift of fluorescence is due to a fast reorganization of the chromophore environment in the excited state (Konold et al., 2014; Faraji and Krylov, 2015; Yoon et al., 2016). Specifically, a direct hydrogen bond between the acylimine oxygen of the chromophore and the E16 carboxyl oxygen of the protein reorganizes to a water-mediated hydrogen bond at the same site. This causes the chromophore to switch from a red emitting form to a far-red emitting form. Since the switch between the two forms occurs on a picosecond time scale, the far-red form dominates in steady-state fluorescence. On the other hand, a small fraction of the far-red form is present in the ground state and can be directly excited (Faraji and Krylov, 2015; Yoon et al., 2016). Our experiment supports this observation because *Ω*, being independent of emission transition dipole moment by definition (Wan and Johnson, 1994), points to the presence of a minor, far-red form in the ground state with a different direction of Δ***μ***. The latter could be assigned to water-mediated hydrogen bonding at the acylimine oxygen. The spectral variation of *Ω* can be used to resolve the contributions of the two forms (we label the red and far-red forms *a* and *b*, respectively), similarly to the case of 1P fluorescence anisotropy measured as a function of excitation wavelength (Lakowicz, 2006). Using the property of additivity of *Ω* and assuming that both forms fluoresce with the same quantum yield *φ*_*b*_ because the red form rapidly converts to the far-red one in the excited state, we can write (Rehms and Callis, 1993):

$$\Omega \left(\lambda \right)=f_{a}\left(\lambda \right)\Omega_{a}+f_{b}\left(\lambda \right)\Omega_{b},$$ (23)

where *f*_a_(*λ*) and *f*_b_(*λ*) are the fractional contributions to the 2PA of the two forms. Specifically, $f_{a}\left(\lambda \right)=\;\frac{\rho_{a}\sigma_{2,a}\left(\lambda \right)}{\rho_{a}\sigma_{2,a}\left(\lambda \right)+\rho_{b}\sigma_{2,b}\left(\lambda \right)}$, and $f_{b}\left(\lambda \right)=\;1-f_{a}\left(\lambda \right)$, where *ρ*_a_ and *ρ*_b_ are the fractional concentrations of forms *a* and *b*. Combining the last relation with Eq. 23, we can write:

$$f_{a}=\frac{\Omega_{b}-\Omega \left(\lambda \right)}{\Omega_{b}-\Omega_{a}},$$ (24)

$$f_{b}=\frac{\text{Ω}\left(\lambda \right)-{\text{Ω}}_{a}}{{\text{Ω}}_{b}-{\text{Ω}}_{a}},$$ (25)

where *Ω*_a_ = 0.698 and *Ω*_b_ = 0.730 are the corresponding pure *Ω* values found experimentally. The spectral contributions of the two forms to the total two-photon absorption spectrum, $\sigma_{2,total}\left(\lambda \right)=\;\rho_{a}\sigma_{2,a}\left(\lambda \right)+\rho_{b}\sigma_{2,b}\left(\lambda \right)$, are calculated as follows:

$$\rho_{a}\sigma_{2,a}\left(\lambda \right)=f_{a}\left(\lambda \right)\sigma_{2,\text{total}}\left(\lambda \right),$$ (26)

$$\;\rho_{b}\sigma_{2,b}\left(\lambda \right)=f_{b}\left(\lambda \right)\sigma_{2,\text{total}}\left(\lambda \right).$$ (27)

and are shown in Figure 6, top, together with the total 2PA spectrum.

Applying the same method to XRFP, we resolved its 2PA spectrum into two forms, Figure 6, bottom. In the case of eqFP670, the independence of *Ω* on excitation wavelength suggests that there is a single ground state conformation in the range of 1,200–1,300 nm. The large Stokes shift can still be tentatively explained by a conformational change of the chromophore’s environment in the excited state.

Table 2 summarizes the magnitude of vector Δ***μ***, and the angles it makes with the vector ***μ*** (*γ*) and the *x*-axis (*δ*), as well as other spectroscopic parameters that we used to obtain this information. For comparison, the table also includes the calculated data for the chromophore in vacuum and relevant literature data. For mPlum and XRFP, where two forms were found in the absorption spectra, we provide a set of parameters for each form. The |Δ***μ***| values were previously measured using Stark spectroscopy for DsRed and mPlum in frozen water/glycerol solution, up to a constant local field factor *f* (Lounis et al., 2001; Abbyad et al., 2007). Both of them are consistent with our data if we assume *f* = 1.75, which is in the range predicted in (Fried et al., 2013) for the frozen solutions, such as those used in Stark measurements. The angle *γ* = 13^0^ found for DsRed in (Lounis et al., 2001) using Stark spectroscopy is close to our value (9^0^). Our experimental value of |Δ***μ***| for DsRed2 also agrees well with the quantum mechanical calculations (List et al., 2012) where different models were explored to describe protein environment. The |Δ***μ***| values obtained for the red anionic chromophore in vacuum (Nifosí et al., 2007; List et al., 2012) with DFT calculations are lower than our semi-empirical calculation probably because the DFT often underestimates |Δ***μ***| (Jacquemin, 2016).

### Electric Fields in RFPs

**FIGURE 7:**
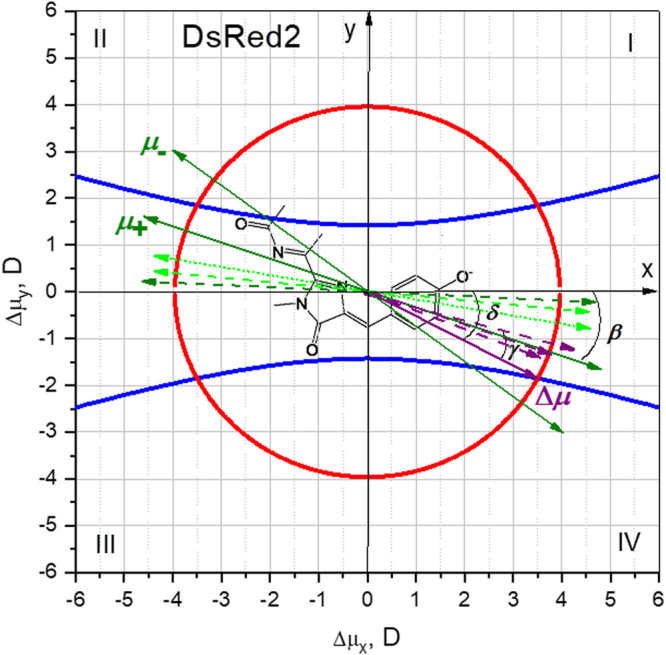
Graphical solution of Eq. 16 (blue curves) and Eq. 21 (red curve), to find the Δ*μ*_x_ and Δ*μ*_y_ components of Δ***μ*** for DsRed2. The structure of the DsRed2 chromophore is displayed in the background, illustrating the molecular system of coordinates. Green dashed bi-directional arrows depict the direction of the oscillating transition dipole moment ***μ***, calculated in (List et al., 2012), for the chromophore in vacuum (light green) and in DsRed protein (dark green). The dotted light green arrow corresponds to a direction of ***μ*** calculated in vacuum in (Ansbacher et al. 2012). Purple dashed arrows correspond to the quantum mechanically calculated Δ***μ*** vector in DsRed protein environment described by three different models (List et al., 2012); see text. The purple solid arrow is the selected Δ***μ*** solution of Eqs 16, 21. It was selected based on the known Δ***μ*** and ***μ*** vectors calculated in (List et al., 2012) and our MD simulations of the electric field; see text. Green solid bi-directional arrows represent the two possible directions of the ***μ*** vector based on our measurement of *γ* and the selected direction of Δ***μ***.

A graphical representation of the curves described by Eqs 16, 21 in the Δ*μ*_x_, Δ*μ*_y_ plane provides the solutions of this system of equations as the intersections of these two curves. Figure 7 plots the equations of the hyperbola Eq. 15 and the circle Eq. 21 for the DsRed2 protein, using the isolated chromophore parameters calculated in Section “*Parameters of the Isolated RFP Chromophore Calculated Quantum Mechanically*” and the experimental values of $\bar{\nu }\;$ and |Δ***μ***| taken from Table 2. There are four possible solutions for Δ***μ*** found at the intersections of the red and blue curves in each of the quadrants I–IV. To resolve this ambiguity, we take into consideration the following two points. 1) The Δ***μ*** vector in DsRed protein was calculated (List et al., 2012) for a polarizable environment (PE-QM), a frozen polarizable environment (FPE) and a non-polarizable environment (NPE). The purple dashed arrows in Figure 7 correspond to these three cases. The angle between Δ***μ*** and the *x*-axis, calculated clockwise, increases in the order: PE-QM, FPE, and NPE, respectively. All three vectors are found in quadrant IV and are close to our experimental result obtained for quadrant IV (purple solid arrow). 2) The direction of the oscillating transition dipole moment ***μ*** was also calculated in (List et al., 2012), both for chromophore in vacuum and in protein with different models describing the environment. In all cases, the ***μ*** vector fell within the quadrants II and IV and made an angle *β* = −(3–9)° with the *x*-axis, Figure 7, showing that the protein environment did not affect it much. The direction of ***μ*** in an isolated RFP chromophore, optimized in vacuum, was also calculated in (Ansbacher et al., 2012) with a result similar to (List et al., 2012), *β* = 10°, Figure 7.

Knowing the direction of Δ***μ*** in a protein and angle *γ*, we can predict the direction of ***μ*** from our own experiments. Since *Ω* measures only an absolute value of *γ*, there will be two possible directions of ***μ*** for each pair of quadrants: I-III and II-IV. Suppose that Δ***μ*** lies within either quadrants II or IV, then two solutions for ***μ***, ***μ***_**+**_ and ***μ***_**−**_ would be possible (Figure 7). The direction of ***μ***_**+**_ agrees better with the calculations of (List et al., 2012; Ansbacher, et al., 2012). Solutions corresponding to quadrants I and III result in worse agreement with theoretically predicted *β*. Therefore, all the evidence presented above suggests that the Δ***μ*** vector most probably falls within quadrant IV, as shown in Figure 7. Its numerical components (Δ*μ*_*x*_ and Δ*μ*_*y*_) are presented in Table 3. Note that the direction of Δ***μ*** corresponds to a flow of electronic density globally from P to I and further to A.

**Table 3:** Components of Δ***μ*** vector and corresponding electric fields for a series of RFPs.

System	$\Delta \mu_{x}$ D	$\Delta \mu_{y}$ D	*E*_*x*_, MV/cm		*E*_*y*_, MV/cm	
			exper.	calc.	exper.	calc.
DsRed2	3.50	−1.85	9.9 ± 0.4	18 ± 3	57 ± 2	61 ± 9
mCherry pH 11.4	2.89	−1.87	12.9 ± 0.5	8 ± 5	57 ± 2	68 ± 15
mScarlet	2.41	−1.91	15.3 ± 0.6	—	55 ± 2	
XRFP (red-shifted)	2.49	−2.40	14.9 ± 0.6	—	33.5 ± 1.3	
XRFP (main)	2.97	−2.06	12.5 ± 0.5	—	48.3 ± 1.9	
mCherry pH 7.4	1.79	−2.20	18.3 ± 0.7	15 ± 2	42 ± 1.7	40 ± 18
mPlum (red-shifted)	1.52	−2.43	19.6 ± 0.8	—	30.2 ± 1.2	—
mPlum (main)	1.73	−2.15	18.6 ± 0.7	36 ± 2	44.3 ± 1.8	88 ± 24
eqFP670 (IV abs)	2.54	−2.52	14.3 ± 0.6		28.3 ± 1.1	
(II abs)	−2.54	2.52	39.5 ± 1.6		247 ± 10	
(III abs)	−2.54	−2.52	39.5 ± 1.6		28.3 ± 1.1	

Electric field components are obtained using the “quasi-empirical” model (see text) (exper.) and using MD calculations (calc.).

The dipole vector diagrams for other proteins (similar to Figure 7) are shown in Supplementary Figure S1. It is interesting to note that the direction of ***μ*** in mCherry (*β* = −8.70°) was recently measured independently in crystal (Myskova et al., 2020) and it is very close to what was predicted theoretically for the isolated chromophore (*β* = -(6÷10°) (List et al., 2012; Ansbacher, et al., 2012) and for the DsRed protein (*β* = −(3–6)°) (List et al., 2012). The *β* angles obtained in liquid solution with our model for ***μ***_**+**_ (*β* = −19° for DsRed2 and −28° for mCherry pH 7.4) are close, but systematically larger in absolute value than those either calculated or measured in crystals. We think that this discrepancy is because the chromophore can visit a larger set of conformations in solution, allowing the acylimine tail to come into better conjugation with the rest of the molecule, thus turning ***μ*** closer to the direction of the tail. Note that the direction of the ***μ*** vector is quite conservative for the whole set of proteins studied here, with values between −19° to −35° (Table 2 and Supplementary Figure S1).

Our next step is to calculate the *E*_*x*_ and *E*_*y*_ components of the electric field from the experimentally defined Δ*μ*_x_ and Δ*μ*_y_, using Eqs 12, 13. The results, that we call “quasi-empirical”, are presented in Table 3 and Supplementary Table S1. To validate our “quasi-empirical” electric fields and to obtain an additional support for the direction of Δ***μ*** vector not only in DsRed but in other RFPs, we performed a series of MD simulations on DsRed, mCherry pH 7.4, mCherry pH 11.4, and mPlum. In this case, electric fields were obtained as negative derivatives of the time-averaged potentials on chromophore atoms along the π-conjugation pathway and projected onto the *x*- or *y*-direction, as was done before for GFPs (Drobizhev et al., 2015). We used the chains of atoms: CD2-CG2-CB2-CA2-C2 for *E*_*x*_ and O2-C2-CA2 for *E*_*y*_ evaluations (see Figure 4 for atoms labeling). The results of these calculations are presented in Supplementary Figure S2 and Supplementary Table S1. Although in some cases the agreement between the experimental and calculated values of the fields is almost quantitative, i.e., within the standard deviations (mCherry pH 7.4 and pH 11.4, if Δ***μ*** is in quadrant IV), in others they agree qualitatively (DsRed and mPlum). Considering the direction of Δ***μ*** in DsRed and mCherry (at pH 7.4 and 11.4), the best match between our “quasi-empirical” model and MD simulations is obtained for Δ***μ*** falling in quadrant IV. For mPlum, the best match corresponds to quadrant III, with quadrant IV being the next closest. If Δ***μ*** of mPlum in fact would lie in quadrant III, the ***μ*** vector would adopt a quite unexpected direction, through quadrants I and III. We consider this improbable because of the “conservation” law for the direction of ***μ***, see (List et al., 2012; Ansbacher, et al., 2012), Table 2, and Supplementary Figure S1. Therefore, we assume that Δ***μ*** of mPlum is within quadrant IV, like in the other three proteins. The components of the Δ***μ*** vector and electric field in all the proteins and in different conformational states, are summarized in Table 3.

### Describing the Rate of TICT Process With Marcus Formalism

We now consider another possible mechanism of fluorescence quenching–ultrafast nonradiative transition (jump) from the excited to the ground state, when the RFP chromophore adopts a twisted conformation at a conical intersection seam. Prior to this event, the chromophore in the excited state proceeds through a slower process of twisting of its phenolate group around the bridging CG2-CB2 bond (Olsen and Smith, 2007; Sun et al, 2012; Moron et al., 2019). According to calculations, the twisting occurs in concert with significant CT across the bridge from the P to the I and A groups. When the angle between P and I rings becomes ∼75°–90°, a whole electronic density of the excited state orbital localizes on the I and A parts (Olsen and Smith, 2007). The charge *q* = 0.32e (e is the electron charge) is transferred from P to A and I in the TICT process (Olsen and Smith, 2007).

TICT is common for many molecules featuring electron-donating and accepting groups capable of rotating relative to each other around a bridging bond (Grabowski et al., 2003). If the final CT state is stabilized by a polar solvent, the fluorescence quantum yield drops dramatically (Grabowski et al., 2003), pointing to the dependence of the CT rate on the thermodynamic free energy difference. We hypothesize that in RFPs, the protein electric field can contribute to stabilization (or destabilization) of the TICT state in RFPs. If, for example, the field directed from I to P (*E*_*x*_) increases (corresponding to concentration of more positive charges on the imidazolinone site and/or more negative charges on the phenyl side in a certain mutant), the CT is expected to accelerate and the conical intersection seam will be reached faster.

In our model, we assume that the potential energy barrier for rotation from a planar to a strongly twisted chromophore is due to steric interactions of the P ring with nearby residues above and below the ring plane. In the RFPs studied here, the most important residues in these positions making direct van der Waals contacts with P are Pro63 (substituted to Thr in eqFP670), Met163 (Lys in DsRed2), and Ile197 (Ala in DsRed2, Arg in mScarlet and eqFP670), as well as Lys70 in DsRed2; see Supplementary Table S3. For our model we assume *a priori* that the barrier is equal for all proteins, i.e., two parabolic potentials corresponding to the initial and final states in the Marcus model always have same stiffness. The electric field, however, changes between proteins, as we have seen.

To correlate the barrier height with a change of free energy, we use Marcus theory formalism. We assume that the rate of TICT in the excited state is a limiting stage for nonradiative relaxation. Therefore, according to the Marcus theory in the high temperature limit, we can write (Sutin, 1999; Bixon and Jortner, 1999):

$$k_{nR\;}=Be^{-\frac{{\left(\Delta G^{0}+\lambda \right)}^{2}}{4\lambda k_{B}T}},$$ (28)

where Δ*G*° is the standard Gibbs free energy change in the CT reaction, *λ* is the reorganization energy, *k*_*B*_ is the Boltzmann factor, and *T* is the absolute temperature. The high temperature limit (*hν*_*V*_ < *k*_*B*_ *T*, where *ν*_*V*_ is the frequency of vibration coupled with CT) can be justified for different low-frequency modes, such as collective vibrations of protein beta-barrel, *ν*_*V*_ = (1.5–9) × 10^11^ s^−1^, (Martin and Matyushov, 2012; Hu et al., 2012; Perticaroli et al., 2014), or twisting modes of the chromophore itself, (3–6) × 10^12^ s^−1^ (Martin et al., 2004). The pre-exponential factor *B* for a single low-frequency mode *ν*_*V*_ can be presented as *B* = κ*ν*_*V*_, where κ is the Landau–Zener dimensionless transmission coefficient (Bixon and Jortner, 1999). If *κ* << 1, the process is considered non-adiabatic, and if *κ* = 1, it is adiabatic. In the non-adiabatic limit,

$$B=\left(\frac{4\pi^{2}}{h}\right)V^{2}{\left(4\pi k_{B}T\lambda \right)}^{-1/2},$$ (29)

where *V* is the electronic coupling parameter. Note that Eqs 28, 29 can be derived independently (of Marcus theory) on the basis of the more general Fermi golden rule for nonradiative electron-vibrational transitions when only low-frequency modes are involved (Bixon and Jortner, 1999; Callis and Liu, 2004).

Taking the logarithm of both sides in Eq. 28, we obtain

$$\ln\left(k_{nR\;}\right)=\ln B-\frac{{\left(\Delta G^{0}+\lambda \right)}^{2}}{4\lambda k_{B}T}.$$ (30)

Suppose that a negative point charge of an absolute value *q* is transferred by a distance Δ*x* opposite to the *x*-axis direction and a distance Δ*y* opposite to the *y*-axis direction, see Figure 4 for definition of *x* and *y* directions. For simplicity, we consider a point charge moving from the geometric center of the initial charge distribution on P (corresponding to a locally excited state) to the geometrical center of the final charge distribution on I and A (corresponding to a TICT state). We assume that the parameters *q*, Δ*x*, and Δ*y* are the same for all the proteins. If an electric field ***E*** = (*E*_x_, *E*_y_) is present in course of the charge transfer, the electrostatic potential energy of the system will change by

$$\Delta U=-q\Delta xE_{x}-q\Delta yE_{y}=-q\Delta x\left(E_{x}+\eta E_{y}\right),\;\;\text{where }\eta =\Delta y/\Delta x\;.$$ (31)

We assume that the field does not change during the process of charge transfer and is the same as that found in previous section. The total free energy change, Δ*G* ^*0*^, consists of a pure molecular (vacuum) part, Δ*G* ^*0*^_vac_, and an electrostatic part (imposed by the protein) Δ*U*:

$$\text{Δ}G^{0}=\text{Δ}G_{vac}^{0}-q\text{Δ}x\left(E_{x}+\eta E_{y}\right).$$ (32)

Substituting this into Eq. 30, we obtain

$$\ln k_{nR\;}=b_{0}+b_{1}\left(E_{x}+\eta E_{y}\right)+b_{2}{\left(E_{x}+\eta E_{y}\right)}^{2},$$ (33)

which is a second-order polynomial in terms of $E_{x}+\eta E_{y}$. In Eq 33, the constant parameters are defined as follows:

$$b_{0}=\ln B-\frac{\lambda }{4k_{B}T}-\frac{\Delta G_{vac}^{0}}{2k_{B}T}-\frac{{\left(\Delta G_{vac}^{0}\right)}^{2}}{4\lambda k_{B}T},$$ (34)

$$b_{1}=\;\frac{q\Delta x}{2k_{B}T}\left[1+\frac{\Delta G_{vac}^{0}}{\lambda }\right],$$ (35)

$$b_{2}=-\frac{{\left(q\Delta x\right)}^{2}}{4\lambda k_{B}T}$$ (36)

**FIGURE 8:**
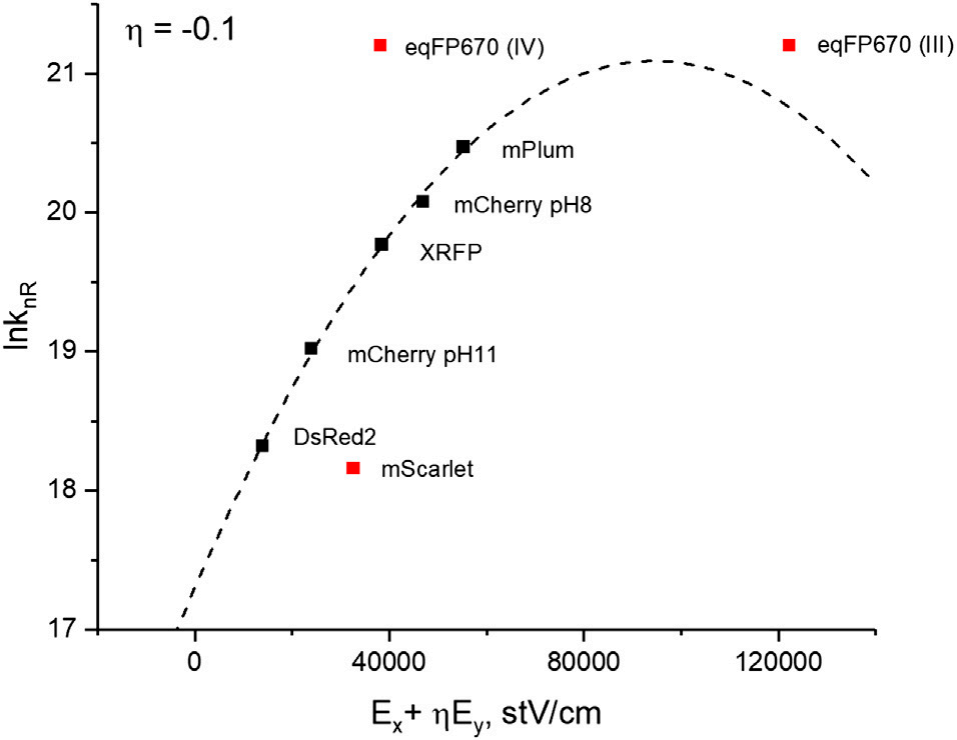
Dependence of nonradiative decay rate (in logarithmic scale) on the change of electrostatic potential energy normalized to the product of charge displacement along *x*-direction (Δ*x*) and the value of charge (*q*). Theoretical fit of the model function Eq. 33 to the data obtained for five proteins (black symbols) is shown by dashed line. mScarlet and eqFP670 data points (red squares) are not included in the fit (see text). Red square labeled eqFP670 (IV) correspond to the electric field calculated from the Δ***μ*** position in quadrant IV, and eqFP670 (III)—to Δ***μ*** position in quadrant III.

Using our experimental results, we plotted ln*k*_*nR*_ vs. $\left(E_{x}+\eta E_{y}\right)$, varying the parameter *η* from −0.2 to 0.1 (Supplementary Figure S3). Out of seven proteins, five (DsRed2, mCherry pH 7.4, mCherry pH 11.4, and red-shifted forms of XRFP and mPlum) fit satisfactorily to a second order polynomial in this range of *η*. mScarlet and eqFP670 drop significantly out of the correlation and therefore we do not include them in the fit (see discussion below). For each *η* value, the residual sum of squares of the fit was calculated and the minimum was achieved at *η* = −0.1 (Supplementary Figure S4). The final plot of  ln*k*_*nR*_ vs $\left(E_{x}+\eta E_{y}\right)$ corresponding to *η* = −0.1 is shown in Figure 8.

In their consideration of TICT, Olsen and Smith calculated the parameters *G* ^*0*^_vac_ and *q* for the isolated RFP chromophore; see Figure 7 of (Olsen and Smith, 2007). We use these values as well as three experimental coefficients, *b*_0_, *b*_1_, and *b*_2_, corresponding to the best fit of our data to the Marcus model (Figure 8) to find the final three parameters: *λ*, Δ*x*, and *B* from the system of Eqs 34–36. The transversal displacement Δ*y* was then calculated according to Δ*y* = *η* Δ*x*. All the parameters are reported in Table 4. (Other Δ*x* and Δ*y* values calculated from the best fits for different *η* values are presented in Supplementary Table S2) Note that the found *B* factor (Table 4) is much smaller than the vibrational frequencies *ν*_*V*_ mentioned above. This suggests non-adiabatic character of CT (*κ* << 1) and justifies applicability of Eq 28. It is interesting that the obtained charge displacement Δ*x* = 6.7 Å is close to the actual chromophore size and to the displacements predicted theoretically in (Olsen and Smith, 2007; Moron et al., 2019). The positive sign of Δ*x* supports the prediction that the electronic density is moving from P to I, and the negative sign and magnitude of Δ*y* (−0.67 Å) suggests a further slight shift from I to A, in qualitative agreement with predictions (Olsen and Smith, 2007).

**TABLE 4:** TICT parameters of the RFP chromophore.

*B* s^−1^	Δ*G*_*vac*_ ^a^ kcal/mole	*λ* kcal/mole	*q* ^a^ e.s.u.	Δ*x* Å	Δ*y* Å
1.5 × 10^9^	−7.9	21.9	1.54 × 10^−10^	6.7	−0.67

^a^ Calculated in (Olsen and Smith, 2007). See text for definitions of parameters.

To estimate more accurately the theoretically predicted magnitude of charge displacement (Olsen and Smith, 2007), we first notice that a part of the LUMO density, localized on the phenolate group before the transfer, is centered close to the middle point between the CE1 and CE2 atoms (Gross et al., 2000; Mochizuki et al., 2007; Hasegawa et al., 2010; List et al., 2012). Furthermore, the electronic density in a 90^0^ P-twisted conformation is delocalized between I and A groups (Olsen and Smith, 2007), with a much higher weight on I than on A (Moron et al., 2019). Therefore, we can assume that the charge density moves from the point between the CE1 and CE2 atoms (whose position does not move during the P rotation) to the C1 atom of imidazolinone. The corresponding crystallographic values (see, e.g., 2H5Q pdb structure for mCherry) in this case are Δ*x* = 6.1 Å and Δ*y* = −0.9 Å, both in good agreement with our findings (Table 4). These results provide strong independent support both for our approach of finding protein electric fields and for describing the field effect in the framework of TICT. Although five proteins of the series fit quite well to the theoretical model, mScarlet and eqFP670 show significant discrepancies.

#### Special Case: mScarlet

In the case of mScarlet, the experimental nonradiative rate is much slower than what is expected from the values of the field. This can be explained by the very tight surrounding of the chromophore that strongly elevates the steric energy barrier for P rotation (Bindels et al., 2017). More specifically, instead of the flexible, neutral Ile197 in mFruits, mScarlet has an Arg residue that faces the phenolate ring and makes multiple van der Waals contacts with it, impeding the P-rotation. Moreover, the Arg side group in mScarlet makes a cobweb of numerous hydrogen bonds that holds it more strongly in place compared to Ile in mFruits and Ala in DsRed2. In the original publication (Bindels et al, 2017), these strong local interactions were invoked to explain the exceptionally planar structure of mScarlet chromophore observed in crystal.

#### Special Case: eqFP670

In the case of eqFP670, the fluorescing state most probably corresponds to a different conformation of the chromophore/environment in the excited state compared to the ground state, see Section “Finding Dipole Moment Difference Δ*μ* and the Angle *γ* between the Vectors *μ* and Δ*μ*”. Unfortunately, this conformation cannot be interrogated with 2PA (in contrast to mPlum and XRFP), because it is not present in the ground state. Therefore, we can only speculate about the values of the electric field corresponding to this conformer. Suppose that Δ***μ*** and the field in the excited state are not very different from the ground state, and that the Δ***μ*** vector lies in quadrant IV (Supplementary Figure S1). Then the nonradiative rate turns out to be several times too fast compared to the expected value based on the model fit (left red square in Figure 8).

However, one cannot rule out the possibility that the Δ***μ*** of eqFP670 occupies another quadrant. For instance, the direction of Δ*μ*_*x*_ can flip if *E*_*x*_ reaches some critical value. This flipping can theoretically occur at $E_{x}^{*}=-\text{Δ}\mu_{0,x}/\text{Δ}\alpha_{xx}=27\text{ MV}/\text{cm}$; see Eq. 10. The *k*_nR_ value steadily increases in concert with *E*_*x*_ in the series of DsRed2, mCherry pH 11.4, XRFP, mCherry pH 7.4, and mPlum (Table 3). Therefore, one would expect an even larger *E*_*x*_ value for eqFP670 than for mPlum (i.e., >19 MV/cm), instead of the predicted *E*_*x*_ = 14 MV/cm (if Δ***μ*** is in quadrant IV). If this were true, it could lead to a negative Δ*μ*_*x*_. This situation would correspond to a position of Δ***μ*** in quadrants II or III with *E*_*x*_ = 39.5 MV/cm for both cases. Choosing between quadrants II and III depends on the direction of Δ*μ*_*y*_. If Δ*μ*_*y*_ > 0, i.e., Δ***μ*** is flipped relatively to its direction in IV and lies in quadrant II, then the predicted *E*_*y*_ value becomes very large (∼250 MV/cm) compared to the rest of the proteins (30–60 MV/cm). This is difficult to explain considering that the electrostatic environment in eqFP670 is not so different from the others. (Estimations show that an additional positive Arg197 and a conserved, presumably negative Glu215, can explain some increase in *E*_*y*_ value of eqFP670 versus mFruits, but not larger than up to a factor of 2.) Therefore, we assume that a position of Δ***μ*** in quadrant III is possible, and we show a data point for eqFP670 corresponding to this quadrant in Figure 8 (right red square). This point is reasonably close to the model fit and provides *E*_*x*_ = 39.5 MV/cm and *E*_*y*_ = 28.3 MV/cm.

Mechanistically, a large value of *E*_*x*_ in eqFP670 (relative to other RFPs) can tentatively be explained by the presence of the Asn143 residue close to the phenolate oxygen of the chromophore. As the crystal structure (4EDS pdb) shows, the carbonyl oxygen atom OD1 of the Asn143 side chain comes in unusually close contact to the phenolate oxygen (∼2.4 Å), which is shorter than the sum of their van der Waals radii. A hydrogen bonding network built around the chromophore phenolate and the Asn143 residue probably helps to hold the two electronegative atoms in such a close contact. Since the Asn carbonyl oxygen carries a partial negative charge, this can result in pushing the negative charge of the phenolate oxygen toward other parts of the chromophore (e.g., I or A). This effect is analogous to an increase of the effective electric field in the *x*-direction that potentially flips the sign of Δ*μ*_*x*_.

## Discussion

It was suggested on theoretical grounds that the nonradiative relaxation of the red FP chromophore in vacuum (Olsen and Smith, 2007) or in proteins (Sun et al., 2012; Moron et al., 2019) generally proceeds via TICT with the phenolate ring turning from 0° to ∼75°–90° around the adjacent C–C bridging bond. This process leads to a conical intersection of the S_1_ and S_0_ potential energy surfaces that opens a doorway for almost instantaneous S_1_ ∼> S_0_ relaxation. The phenolate rotation is barrierless for chromophore in vacuum, but encounters a potential barrier in proteins due to steric interactions between the phenolate ring and few nearby amino acid side chains. Since the twisting is coupled to significant charge transfer in the excited state, an external (to the chromophore) electric field could either facilitate or hinder this process, depending on its strength and direction. We apply the Marcus formalism to correlate the barrier height and the free energy difference (Δ*G* ^0^) between the final and initial states of the charge transfer. In this model, the Δ*G* ^0^ contains a contribution equal to the change of electrostatic potential energy between the two states. We observe that for five out of seven proteins, including DsRed2, mCherry (pH 7.4 and pH 11.4), mPlum, and XRFP, the radiationless relaxation rate follows the Marcus theory.

For mScarlet, the measured nonradiative rate is much slower than predicted by the model. We explain this by a much higher potential barrier for rotation of phenolate because of steric clashes with the R198 and Pro64 side chains. The former occupies a unique position on top of the phenolate group and makes several van der Waals contacts with it. The second flanks the phenolate from the opposite side and due to its rigid nature provides an additional barrier for P-rotation. Therefore, we can conclude that a combination of these two amino acids in the chromophore pocket is probably a main factor securing high brightness of mScarlet, compared to, e.g., XRFP (experiencing similar electric field, Figure 8).

In contrast, the nonradiative rate of eqFP670 appears to be larger than predicted by our model, for both possible directions of Δ***μ***, i.e. in quadrants III and IV, Figure 8. Although field-facilitated TICT can qualitatively explain the very fast nonradiative rate of eqFP670 (see previous section), other mechanisms cannot be ruled out. One is vibrational relaxation, described by the energy gap law (“*Checking the Energy Gap Law*” section). For a far-red emitting protein such as eqFP670, this mechanism can strongly contribute to an increase of *k*_nR_. Another possibility is that some other molecular parameters involved in TICT are different in eqFP670 compared to other RFPs. These include the charge *q*, and CT distances Δ*x* or Δ*y*, as well as the potential barrier to rotation. Differences in any of the first three are probable, especially if the acylimine tail adopts a different conformation during the process of excited state relaxation similar to what was suggested in mPlum (Moron et al., 2019). There are two different factors that could affect the height of potential barrier for P-rotation. First, similar to mScarlet, the phenolate of eqFP670 is flanked by Arg197 with four van der Waals contacts between them (although in mScarlet there are six). Also, Arg197 participates in a number of hydrogen bonds that hold its position fixed. On the other hand, the more flexible Thr60 found on the opposite side of the phenolate group in eqFP670, replacing the rigid cycle of the Pro63 residue, probably makes phenolate rotation easier.

**FIGURE 9:**
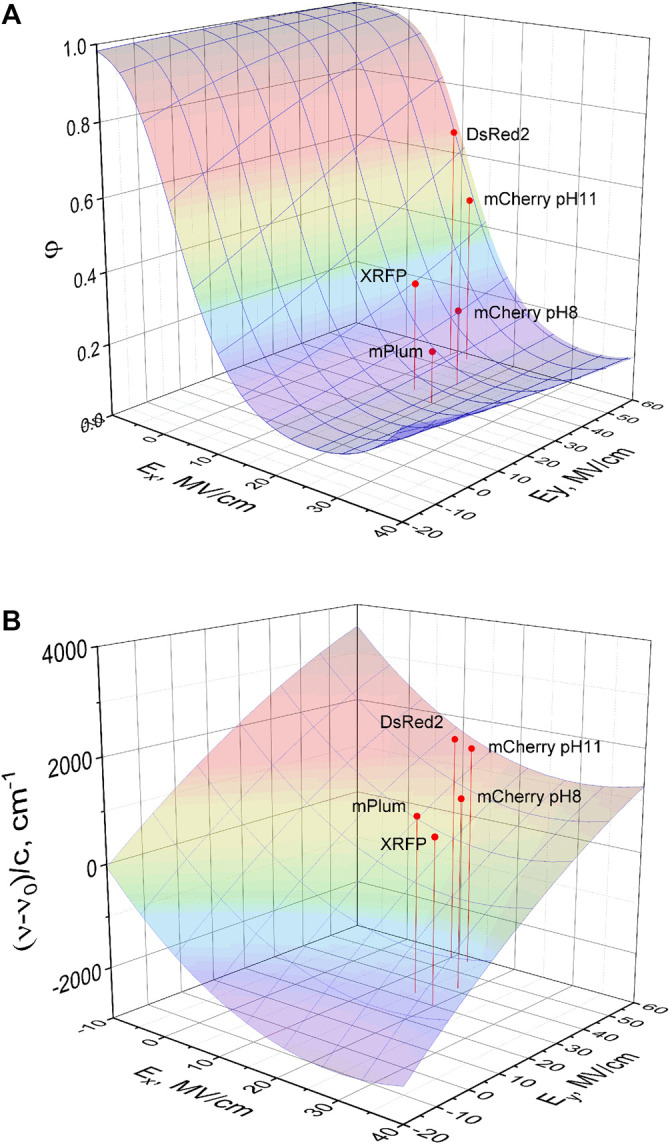
3D plot representing quantum yield **(A)** and shift of peak absorption transition frequency relative to the vacuum frequency **(B)** as a function of *E*_*x*_ and *E*_*y*_ in a set of RFPs with a “moderately-soft” phenolate pocket (see text). Five representative RFPs are shown by red dots. Large quantum yields in **(A)** correspond to red and small quantum yields—to purple color of the surface. The calculation is based on model Eqs 2, 33–36 and model parameters presented in Table 4. Large negative shifts in **(B)** corresponds to purple and large positive shifts—to red surface color. The prediction is based on Eq. 15 and parameters from “*Parameters of the Isolated RFP Chromophore Calculated Quantum Mechanically*” section.

If we only consider the RFPs with a “moderately-soft” internal pocket around phenolate group, i.e., similar to those containing Pro63 and Ile197 (or Ala197), we can establish some qualitative structure-property relationships for photophysical parameters that can potentially help to guide future RFP engineering. Figure 9A shows a 3D plot of the quantum yield as a function of the electric field components *E*_*x*_ and *E*_*y*_. The quantum yield is calculated using Eq. 2 with *k*_R_ = 0.158 × 10^9^ s^−1^ (mean of all seven *k*_R_ values, Table 1), and *k*_nR_ described by Eqs 33–36. As one can see, for each fixed *E*_*y*_ value, the quantum yield first decreases as *E*_*x*_ increases, then reaches a minimum, and then starts to increase. For all reasonable *E*_*y*_ values, in the region of negative *E*_x_ values, the potential barrier for TICT via P-rotation becomes very high due to the combined action of the “charge locking” effect of *E*_*x*_ and steric clashes of phenolate with nearby residues. This results in *φ* = 1.

With increasing positive *E*_*x*_, the barrier becomes lower, thus accelerating nonradiative relaxation. The *k*_nR_ reaches its maximum (minimum φ) when Δ*G* ^0^ = −*λ*, in accord with the Marcus model. Even larger *E*_*x*_ values correspond to a so-called inverted Marcus region (Bixon and Jortner, 1999), where the *k*_nR_ rate starts to decrease again and quantum yield to increase. For a fixed *E*_*x*_ within the normal Marcus region, φ increases with the increase of *E*_*y*_, but the absolute values of gradients are much smaller than for *E*_*x*_. This behavior follows from the fact that the charge is transferred counter to the *E*_*y*_ field, but at a much shorter distance compared to the *x*-direction. For an illustration, we show the data points corresponding to DsRed2, mCherry pH 11.4, mCherry pH 7.4, mPlum, and XRFP. The transition from DsRed2 to mCherry pH 11.4 corresponds to the drop of quantum yield by 30%, following an increase of *E*_*x*_ by 3 MV/cm (with *E*_*y*_ unchanged), whereas transition from mCherry pH 7.4 to mPlum also corresponds to a drop of *φ* by 30%, but with the decrease of *E*_*y*_ by 12 MV/cm (with almost constant *E*_*x*_ value).

The absorption frequency shift (relatively to vacuum ${\bar{\nu }}_{0}$ = 16,260 cm^−1^, λ_abs,0_ = 615 nm) as a function of *E*_*x*_ and *E*_*y*_ is presented in Figure 9B. It is calculated according to Eq. 14. Here, the further red-shifted variants correspond to small positive (or, better, even negative) *E*_*y*_ values simultaneously with *E*_*x*_ values around 20–30 MV/cm. To have both a red-shifted absorption and a reasonably high quantum yield, one has to keep both *E*_*x*_ and *E*_*y*_ in the range of small positive or negative values, i.e., from −10 to +10 MV/cm. A favorably low *E*_*x*_ of the DsRed2 protein leading to a high quantum yield is most likely due to the positively charged Lys163 residue hydrogen-bonded to the phenolate oxygen. In mFruits and mScarlet, *E*_*x*_ is larger, probably because of lacking of the charge in that position.

Possible mutagenesis strategy for optimizing both parameters (*φ* and *λ*_abs_) using the DsRed scaffold could contain the following steps. First, one should keep a positively charged residue H-bonded to the phenolate oxygen (to have a small *E*_*x*_). Second, one should concentrate more positive charges close to the acylimine oxygen (to decrease *E*_*y*_) and/or more negative charge on the imidazolinone carbonyl oxygen (to further decrease *E*_*x*_ and decrease *E*_*y*_). The last change may be a difficult task however, because the Arg95 residue hydrogen bonding to the imidazolinone oxygen seems to be conservative in RFPs. It is interesting that similar recipes for improving quantum yield and shifting fluorescence to the red were put forward on the basis of quantum calculations of several different mPlum conformers (Moron et al., 2019).

**FIGURE 10:**
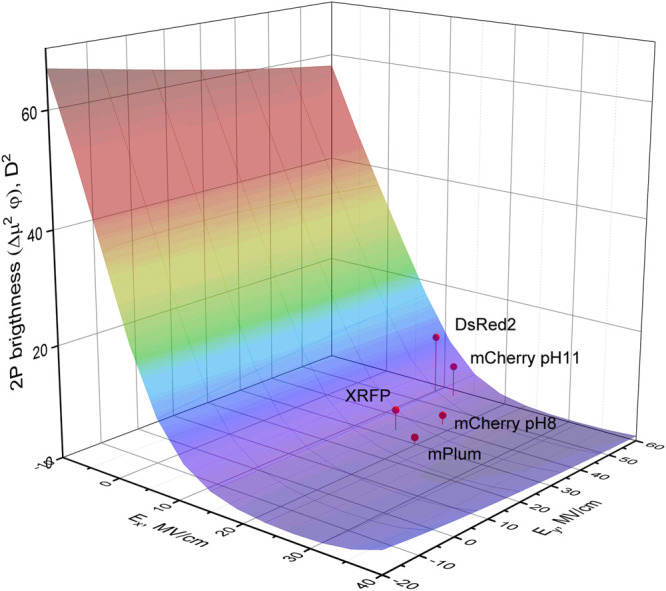
3D plot representing the 2P brightness (roughly proportional to Δ***μ*** ^2^ *φ*) in the region of the 0–0 excitation frequency as a function of *E*_*x*_ and *E*_*y*_. Large brightness corresponds to red color on the surface and small brightness—to purple. Four representative RFPs are shown by red dots.

We finally would like to note that in the case of two-photon excitation, peak absorption wavelengths of RFPs automatically fall in the tissue transparency window and therefore the 2P brightness, but not the position of 2PA peak becomes a most critical parameter. The relative 2P brightness can be estimated to a first approximation as a product Δ***μ*** ^2^ *φ*. Both Δ***μ*** ^2^ and *φ* depend on the electric field and our model, see Figure 10, predicts that for getting 2P brighter variants one has to decrease or even flip the sign of *E*_*x*_ component, with *E*_*y*_ playing not too critical role. The structural guidelines for increasing 2P brightness are therefore similar to what was described above for obtaining higher quantum yield.

## Data Availability

The original contributions presented in the study are included in the article/Supplementary Material, further inquiries can be directed to the corresponding author.

## References

[B1] Abbyad P. Childs W. Shi X. Boxer S. G. (2007). Dynamic Stokes shift in green fluorescent protein variants. Proc. Natl. Acad. Sci. U.S.A. 104 (51), 20189–20194. 10.1073/pnas.0706185104 18077381 PMC2154406

[B2] Afgan E. Baker D. Batut B. van den Beek M. Bouvier D. Cech M. (2018). The Galaxy platform for accessible, reproducible and collaborative biomedical analyses: 2018 update. Nucleic Acids Res. 46, W537–W544. 10.1093/nar/gky379 29790989 PMC6030816

[B3] Altoe P. Bernardi F. Garavelli M. Orlandi G. Negri F. (2005). Solvent effects on the vibrational activity and photodynamics of the green fluorescent protein chromophore: a quantum-chemical study. J. Am. Chem. Soc. 127 (11), 3952–3963. 10.1021/ja0451517 15771532

[B4] Ansbacher T. Srivastava H. K. Stein T. Baer R. Merkx M. Shurki A. (2012). Calculation of transition dipole moment in fluorescent proteins–towards efficient energy transfer. Phys. Chem. Chem. Phys. 14 (12), 4109–4117. 10.1039/c2cp23351g 22331099

[B5] Atkins P. W. Friedman R. S. (1997). Molecular Quantum Mechanics. Oxford, New York, Tokyo: Oxford University Press.

[B6] Berlin Y. Burin A. Friedrich J. Köhler J. (2006). Spectroscopy of proteins at low temperature. Part I: Experiments with molecular ensembles. Phys. Life Rev. 3 (4), 262–292. 10.1016/j.plrev.2006.09.001

[B7] Bindels D. S. Haarbosch L. van Weeren L. Postma M. Wiese K. E. Mastop M. (2017). mScarlet: a bright monomeric red fluorescent protein for cellular imaging. Nat. Methods 14 (1), 53–56. 10.1038/nmeth.4074 27869816

[B8] Bixon M. Jortner J. (1999). “Electron transfer - from isolated molecules to biomolecules,” in Electron Transfer–From Isolated Molecules to Biomolecules. Pt. 1. Editor Jortner J. Bixon M. (New York: Wiley), 35–203.

[B9] Bloebmergen N. (1965). Nonlinear Optics. New York: Benjamin.

[B10] Boens N. Qin W. Basarić N. Hofkens J. Ameloot M. Pouget J. (2007). Fluorescence lifetime standards for time and frequency domain fluorescence spectroscopy. Anal. Chem. 79 (5), 2137–2149. 10.1021/ac062160k 17269654 PMC6816264

[B11] Callis P. R. Burgess B. K. (1997). Tryptophan fluorescence shifts in proteins from hybrid simulations: an electrostatic approach. J. Phys. Chem. B 101 (46), 9429–9432. 10.1021/jp972436f

[B12] Callis P. R. Liu T. (2004). Quantitative prediction of fluorescence quantum yields for tryptophan in proteins. J. Phys. Chem. B 108 (14), 4248–4259. 10.1021/jp0310551

[B13] Chudakov D. M. Feofanov A. V. Mudrik N. N. Lukyanov S. Lukyanov K. A. (2003). Chromophore environment provides clue to “kindling fluorescent protein” riddle. J. Biol. Chem. 278 (9), 7215–7219. 10.1074/jbc.M211988200 12496281

[B14] Chudakov D. M. Matz M. V. Lukyanov S. Lukyanov K. A. (2010). Fluorescent proteins and their applications in imaging living cells and tissues. Physiol. Rev. 90 (3), 1103–1163. 10.1152/physrev.00038.2009 20664080

[B15] Cramer L. E. Spears K. G. (1978). Hydrogen bond strengths from solvent-dependent lifetimes of Rose Bengal dye. J. Am. Chem. Soc. 100 (1), 221–227. 10.1021/ja00469a039

[B16] Day R. N. Davidson M. W. (2009). The fluorescent protein palette: tools for cellular imaging. Chem. Soc. Rev. 38 (10), 2887–2921. 10.1039/b901966a 19771335 PMC2910338

[B17] Dmitrienko D. V. Vrzheshch E. P. Drutsa V. L. Vrzheshch P. V. (2006). Red fluorescent protein DsRed: Parametrization of its chromophore as an amino acid residue for computer modeling in the OPLS-AA force field. Biochem. Mosc. 71 (10), 1133–1152. 10.1134/s0006297906100129 17125463

[B18] Drobizhev M. Tillo S. Makarov N. S. Hughes T. E. Rebane A. (2009). Color hues in red fluorescent proteins are due to internal quadratic Stark effect. J. Phys. Chem. B 113 (39), 12860–12864. 10.1021/jp907085p 19775174 PMC2893592

[B19] Drobizhev M. Makarov N. S. Tillo S. E. Hughes T. E. Rebane A. (2011). Two-photon absorption properties of fluorescent proteins. Nat. Methods 8 (5), 393–399. 10.1038/nmeth.1596 21527931 PMC4772972

[B20] Drobizhev M. Scott J. N. Callis P. R. Rebane A. (2012a). All-optical sensing of the components of the internal local electric field in proteins. IEEE Photon. J. 4 (5), 1996–2001. 10.1109/JPHOT.2012.2221124 PMC4238891 25419440

[B21] Drobizhev M. Makarov N. S. Tillo S. E. Hughes T. E. Rebane A. (2012b). Describing two-photon absorptivity of fluorescent proteins with a new vibronic coupling mechanism. J. Phys. Chem. B 116 (5), 1736–1744. 10.1021/jp211020k 22224830 PMC3280616

[B22] Drobizhev M. Callis P. R. Nifosì R. Wicks G. Stoltzfus C. Barnett L. (2015). Long- and short-range electrostatic fields in GFP mutants: implications for spectral tuning. Sci. Rep. 5, 13223. 10.1038/srep13223 26286372 PMC4541067

[B23] Drobizhev M. Molina R. S. Hughes T. E. (2020). Characterizing the two-photon absorption properties of fluorescent molecules in the 680–1,300 nm spectral range. Bio. Protoc. 10 (2), 33. 10.21769/BioProtoc.3498 PMC7409827 32775539

[B24] Englman R. Jortner J. (1970). The energy gap law for radiationless transitions in large molecules. Mol. Phys. 18 (2), 145–164. 10.1080/00268977000100171

[B25] Faraji S. Krylov A. I. (2015). On the nature of an extended stokes shift in the mplum fluorescent protein. J. Phys. Chem. B 119 (41), 13052–13062. 10.1021/acs.jpcb.5b07724 26402581

[B26] Fleming G. R. Knight A. W. E. Morris J. M. Morrison R. J. S. Robinson G. W. (1977). Picosecond fluorescence studies of xanthene dyes. J. Am. Chem. Soc. 99 (13), 4306–4311. 10.1021/ja00455a017

[B27] Fried S. D. Wang L. P. Boxer S. G. Ren P. Pande V. S. (2013). Calculations of the electric fields in liquid solutions. J. Phys. Chem. B 117 (50), 16236–16248. 10.1021/jp410720y 24304155 PMC4211882

[B28] Frisch M. J. Trucks G. W. Schlegel H. B. Scuseria G. E. Robb M. A. Cheeseman J. R. (2016). Gaussian 09, Revision A.02. Wallingford CT: Gaussian, Inc.

[B29] Geissinger P. Kohler B. E. Woehl J. C. (1995). Electric field and structure in the myoglobin heme pocket. J. Phys. Chem. 99 (45), 16527–16529. 10.1021/j100045a008

[B30] Gibson DG Young L Chuang RY Venter JC Hutchison CA Smith HO (2009). Enzymatic assembly of DNA molecules up to several hundred kilobases. Nat. Methods 6, 343–345. 10.1038/nmeth.1318 19363495

[B31] Grabherr M. G. Haas B. J. Yassour M. Levin J. Z. Thompson D. A. Amit I. (2011). Full-length transcriptome assembly from RNA-Seq data without a reference genome. Nat. Biotechnol. 29, 644–652. 10.1038/nbt.1883 21572440 PMC3571712

[B32] Grabowski Z. R. Rotkiewicz K. Rettig W. (2003). Structural changes accompanying intramolecular electron transfer: focus on twisted intramolecular charge–transfer states and structures. Chem. Rev. 103 (10), 3899–4032. 10.1021/cr940745l 14531716

[B33] Gross L. A. Baird G. S. Hoffman R. C. Baldridge K. K. Tsien R. Y. (2000). The structure of the chromophore within DsRed, a red fluorescent protein from coral. Proc. Natl. Acad. Sci. U.S.A. 97 (22), 11990–11995. 10.1073/pnas.97.22.11990 11050230 PMC17282

[B34] Haas B. J. Papanicolaou A. Yassour M. Grabherr M. Blood P. D. Bowden J. (2013). De novo transcript sequence reconstruction from RNA-seq using the Trinity platform for reference generation and analysis. Nat. Protoc. 8, 1494–1512. 10.1038/nprot.2013.084 23845962 PMC3875132

[B35] Hasegawa J. Y. Ise T. Fujimoto K. J. Kikuchi A. Fukumura E. Miyawaki A. (2010). Excited states of fluorescent proteins, mKO and DsRed: chromophore-protein electrostatic interaction behind the color variations. J. Phys. Chem. B 114 (8), 2971–2999. 10.1021/jp9099573 20131896

[B36] Hu G. Michielssens S. Moors S. L. Ceulemans A. (2012). The harmonic analysis of cylindrically symmetric proteins: a comparison of Dronpa and a DNA sliding clamp. J. Mol. Graph Model 34, 28–37. 10.1016/j.jmgm.2011.12.005 22306411

[B37] Jacquemin D. (2016). Excited-state dipole and quadrupole moments: TD-DFT versus CC2. J. Chem. Theory Comput. 12 (8), 3993–4003. 10.1021/acs.jctc.6b00498 27385324 PMC4980690

[B38] Jung G. Wiehler J. Zumbusch A. (2005). The photophysics of green fluorescent protein: influence of the key amino acids at positions 65, 203, and 222. Biophys. J. 88 (3), 1932–1947. 10.1529/biophysj.104.044412 15613627 PMC1305246

[B39] Jung G. Brockhinke A. Gensch T. Hotzer B. Schwedler S. Koydan Weettil S. (2012). “Fluorescence lifetime of fluorescent proteins,” in Fluorescent Protein 1. From Understanding to Design. Editor Jung G. (Berlin: Springer), 69–97. Ch. 11

[B40] Konold P. Regmi C. K. Chapagain P. P. Gerstman B. S. Jimenez R. (2014). Hydrogen bond flexibility correlates with Stokes shift in mPlum variants. J. Phys. Chem. B 118 (11), 2940–2948. 10.1021/jp412371y 24611679 PMC4084698

[B41] Lakowicz J. R. Laczko G. Gryczynski I. (1986). 2‐GHz frequency‐domain fluorometer. Rev. Sci. Instrum. 57 (10), 2499–2506. 10.1063/1.1139215

[B42] Lakowicz J. R. (2006). Principles of Fluorescence Spectroscopy. 3rd Ed. Berlin: Springer.

[B43] Li J. Williams B. Cramer C. J. Truhlar D. G. (1999). A class IV charge model for molecular excited states. J. Chem. Phys. 110 (2), 724–733. 10.1063/1.478180

[B44] Lin M. Z. McKeown M. R. Ng H. L. Aguilera T. A. Shaner N. C. Campbell R. E. (2009). Autofluorescent proteins with excitation in the optical window for intravital imaging in mammals. Chem. Biol. 16 (11), 1169–1179. 10.1016/j.chembiol.2009.10.009 19942140 PMC2814181

[B45] List N. H. Olsen J. M. Jensen H. J. Steindal A. H. Kongsted J. (2012). Molecular-level insight into the spectral tuning mechanism of the DsRed chromophore. J. Phys. Chem. Lett. 3 (23), 3513–3521. 10.1021/jz3014858 26290981

[B46] Lounis B. Deich J. Rosell F. I. Boxer S. G. Moerner W. E. (2001). Photophysics ofDsRed, a red fluorescent protein, from the ensemble to the single-molecule level. J. Phys. Chem. B 105 (21), 5048–5054. 10.1021/jp010116x

[B47] Magde D. Wong R. Seybold P. G. (2002). Fluorescence quantum yields and their relation to lifetimes of rhodamine 6G and fluorescein in nine solvents: improved absolute standards for quantum yields. Photochem. Photobiol. 75 (4), 327–334. 10.1562/0031-8655(2002)075<0327:fqyatr>2.0.co;2 12003120

[B48] Manas E. S. Vanderkooi J. M. Sharp K. A. (1999). The effects of protein environment on the low temperature electronic spectroscopy of cytochromecand microperoxidase-11. J. Phys. Chem. B 103 (30), 6334–6348. 10.1021/jp9908552

[B49] Martin D. R. Matyushov D. V. (2012). Non-Gaussian statistics and nanosecond dynamics of electrostatic fluctuations affecting optical transitions in proteins. J. Phys. Chem. B 116 (34), 10294–10300. 10.1021/jp305757t 22861814

[B50] Martin M. E. Negri F. Olivucci M. (2004). Origin, nature, and fate of the fluorescent state of the green fluorescent protein chromophore at the CASPT2//CASSCF resolution. J. Am. Chem. Soc. 126 (17), 5452–5464. 10.1021/ja037278m 15113217

[B51] Masters T. A. Marsh R. J. Blacker T. S. Armoogum D. A. Larijani B. Bain A. J. (2018). Polarized two-photon photoselection in EGFP: Theory and experiment. J. Chem. Phys. 148 (13), 134311. 10.1063/1.5011642 29626864

[B52] Matz M. V. Fradkov A. F. Labas Y. A. Savitsky A. P. Zaraisky A. G. Markelov M. L. (1999). Fluorescent proteins from nonbioluminescent Anthozoa species. Nat. Biotechnol. 17 (10), 969–973. 10.1038/13657 10504696

[B53] Meath W. J. Power E. A. (1984). On the importance of permanent moments in multiphoton absorption using perturbation theory. J. Phys. B: At. Mol. Phys. 17, 763–781. 10.1088/0022-3700/17/5/017

[B54] Mochizuki Y. Nakano T. Amari S. Ishikawa T. Tanaka K. Sakurai M. (2007). Fragment molecular orbital calculations on red fluorescent protein (DsRed). Chem. Phys. Lett. 433 (4), 360–367. 10.1016/j.cplett.2006.11.088 19127982

[B55] Molina R. S. Tran T. M. Campbell R. E. Lambert G. G. Salih A. Shaner N. C. (2017). Blue-Shifted green fluorescent protein homologues are brighter than enhanced green fluorescent protein under two-photon excitation. J. Phys. Chem. Lett. 8 (12), 2548–2554. 10.1021/acs.jpclett.7b00960 28530831 PMC5474692

[B56] Molina R. S. Qian Y. Wu J. Shen Y. Campbell R. E. Drobizhev M. (2019). Understanding the fluorescence change in red genetically encoded calcium ion indicators. Biophys. J. 116 (10), 1873–1886. 10.1016/j.bpj.2019.04.007 31054773 PMC6531872

[B57] Moron V. Marazzi M. Wanko M. (2019). Far red fluorescent proteins: where is the limit of the acylimine chromophore? J. Chem. Theory Comput. 15 (7), 4228–4240. 10.1021/acs.jctc.9b00070 31146524

[B58] Myskova J. Rybakova O. Brynda J. Khoroshyy P. Bondar A. Lazar J. (2020). Directionality of light absorption and emission in representative fluorescent proteins. Proc. Natl. Acad. Sci. 117 (51), 32395–32401. 33273123 10.1073/pnas.2017379117 PMC7768707

[B59] Nifosí R. Amat P. Tozzini V. (2007). Variation of spectral, structural, and vibrational properties within the intrinsically fluorescent proteins family: a density functional study. J. Comput. Chem. 28 (14), 2366–2377. 10.1002/jcc.20764 17600852

[B60] Nifosì R. Mennucci B. Filippi C. (2019). The key to the yellow-to-cyan tuning in the green fluorescent protein family is polarisation. Phys. Chem. Chem. Phys. 21 (35), 18988–18998. 10.1039/c9cp03722e 31464320

[B61] O’Hagan W. J. McKenna M. Sherrington D. C. Rolinski O. J. Birch D. J. S. (2001). MHz LED source for nanosecond fluorescence sensing. Measur. Sci. Technol. 13 (1), 84.

[B62] Olsen S. Smith S. C. (2007). Radiationless decay of red fluorescent protein chromophore models via twisted intramolecular charge-transfer states. J. Am. Chem. Soc. 129 (7), 2054–2065. 10.1021/ja066430s 17253685

[B63] Park J. W. Rhee Y. M. (2016). Electric field keeps chromophore planar and produces high yield fluorescence in green fluorescent protein. J. Am. Chem. Soc. 138, 13619–13629. 10.1021/jacs.6b06833 27662359

[B64] Perticaroli S. Nickels J. D. Ehlers G. Sokolov A. P. (2014). Rigidity, secondary structure, and the universality of the boson peak in proteins. Biophys. J. 106 (12), 2667–2674. 10.1016/j.bpj.2014.05.009 24940784 PMC4070067

[B65] Pronk S. Páll S. Schulz R. Larsson P. Bjelkmar P. Apostolov R. (2013). GROMACS 4.5: a high-throughput and highly parallel open source molecular simulation toolkit. Bioinformatics 29 (7), 845–854. 10.1093/bioinformatics/btt055 23407358 PMC3605599

[B66] Reed W. Politi M. J. Fendler J. H. (1981). Rotational diffusion of rose bengal in aqueous micelles: evidence for extensive exposure of the hydrocarbon chains. J. Am. Chem. Soc. 103 (15), 4591–4593. 10.1021/ja00405a056

[B67] Rehms A. A. Callis P. R. (1993). Two-photon fluorescence excitation spectra of aromatic amino acids. Chem. Phys. Lett. 208 (3), 276–282. 10.1016/0009-2614(93)89075-s

[B68] Reisfeld R. Zusman R. Cohen Y. Eyal M. (1988). The spectroscopic behaviour of rhodamine 6G in polar and non-polar solvents and in thin glass and PMMA films. Chem. Phys. Lett. 147 (2), 142–147. 10.1016/0009-2614(88)85073-5

[B69] Ridley J. Zerner M. (1973). An intermediate neglect of differential overlap technique for spectroscopy: Pyrrole and the azines. Theoret. Chim. Acta 32 (2), 111–134. 10.1007/bf00528484

[B70] Rodgers M. A. J. (1981). Picosecond fluorescence studies of rose bengal in aqueous micellar dispersions. Chem. Phys. Lett. 78 (3), 509–514. 10.1016/0009-2614(81)85248-7

[B71] Schäfer L. V. Groenhof G. Klingen A. R. Ullmann G. M. Boggio-Pasqua M. Robb M. A. (2007). Photoswitching of the fluorescent protein asFP595: Mechanism, proton pathways, and absorption spectra. Angew Chem. Int. Ed. Engl. 46 (4), 530–536. 10.1002/anie.200602315 17094157

[B72] Schweitzer-Stenner R. (2008). Internal electric field in cytochrome C explored by visible electronic circular dichroism spectroscopy. J. Phys. Chem. B 112 (33), 10358–66. 10.1021/jp802495q 18665633

[B73] Shaner N. C Steinbach P. A. Tsien R. Y. (2005). A guide to choosing fluorescent proteins. Nat. Methods 2 (12), 905–909. 10.1038/nmeth819 16299475

[B74] Shaner N. C. (2018). “New fluorescent proteins from unexpected sources,” in Janelia research conference fluorescent proteins and biological sensors VI, 7–10. Janelia Research Campus.

[B75] Shcherbo D. Shemiakina I. I. Ryabova A. V. Luker K. E. Schmidt B. T. Souslova E. A. (2010). Near-infrared fluorescent proteins. Nat. Methods 7 (10), 827–829. 10.1038/nmeth.1501 20818379 PMC4425247

[B76] Shu X. Shaner N. C. Yarbrough C. A. Tsien R. Y. Remington S. J. (2006). Novel chromophores and buried charges control color in mFruits. Biochemistry 45 (32), 9639–9647. 10.1021/bi060773l 16893165

[B77] Simine L. Lammert H. Sun L. Onuchic J. N. Rossky P. J. (2018). Fluorescent proteins detect host structural rearrangements via electrostatic mechanism. J. Am. Chem. Soc. 140 (4), 1203–1206. 10.1021/jacs.7b10851 29328673

[B78] Stiel A. C. Andresen M. Bock H. Hilbert M. Schilde J. Schönle A. (2008). Generation of monomeric reversibly switchable red fluorescent proteins for far-field fluorescence nanoscopy. Biophys. J. 95 (6), 2989–2997. 10.1529/biophysj.108.130146 18658221 PMC2527278

[B79] Sun Q. Li Z. Lan Z. Pfisterer C. Doerr M. Fischer S. (2012). Isomerization mechanism of the HcRed fluorescent protein chromophore. Phys. Chem. Chem. Phys. 14 (32), 11413–11424. 10.1039/c2cp41217a 22801745

[B80] Sutin N. (1999). “Electron transfer reactions in solution: A historical perspective,” in Electron Transfer ‐ From Isolated Molecules to Biomolecules. Pt. 1. Editor Jortner J. Bixon M. (New York: Wiley), 7–33.

[B81] Taguchi N. Mochizuki Y. Nakano T. Amari S. Fukuzawa K. Ishikawa T. (2009). Fragment molecular orbital calculations on red fluorescent proteins (DsRed and mFruits). J. Phys. Chem. B 113 (4), 1153–1161. 10.1021/jp808151c 19127982

[B82] Tolbert L. M. Baldridge A. Kowalik J. Solntsev K. M. (2012). Collapse and recovery of green fluorescent protein chromophore emission through topological effects. Acc. Chem. Res. 45 (2), 171–181. 10.1021/ar2000925 21861536

[B83] Tsien R. Y. (1998). The green fluorescent protein, Annu. Rev. Biochem. 67, 509–544. 10.1146/annurev.biochem.67.1.509 9759496

[B84] Vivian J. T. Callis P. R. (2001). Mechanisms of tryptophan fluorescence shifts in proteins. Biophys. J. 80 (5), 2093–2109. 10.1016/S0006-3495(01)76183-8 11325713 PMC1301402

[B85] Wan C. Johnson C. K. (1994). Time-resolved anisotropic two-photon spectroscopy. Chem. Phys. 179 (3), 513–531. 10.1016/0301-0104(94)87027-6

[B86] Wang L. Jackson W. C. Steinbach P. A. Tsien R. Y. (2004). Evolution of new nonantibody proteins via iterative somatic hypermutation. Proc. Natl. Acad. Sci. U.S.A. 101 (48), 16745–16749. 10.1073/pnas.0407752101 15556995 PMC529417

[B87] Ward W. W. (2005). Biochemical and physical properties of green fluorescent protein, Methods Biochem. Anal. 47, 39–65. 10.1002/0471739499.ch3 16335709

[B88] Wiedenmann J. Oswald F. Nienhaus G. U. (2009). Fluorescent proteins for live cell imaging: opportunities, limitations, and challenges. IUBMB Life 61 (11), 1029–1042. 10.1002/iub.256 19859977

[B89] Yanushevich Y. G. Staroverov D. B. Savitsky A. P. Fradkov A. F. Gurskaya N. G. Bulina M. E. (2002). A strategy for the generation of non-aggregating mutants of Anthozoa fluorescent proteins. FEBS Lett. 511 (1–3), 11–14. 10.1016/s0014-5793(01)03263-x 11821040

[B90] Yoon E. Konold P. E. Lee J. Joo T. Jimenez R. (2016). Far-red emission of mPlum fluorescent protein results from excited-state interconversion between chromophore hydrogen-bonding states. J. Phys. Chem. Lett. 7 (12), 2170–2174. 10.1021/acs.jpclett.6b00823 27214167

