# Supplementary material for: Local Electric Field Controls Fluorescence Quantum Yield of Red and Far-Red Fluorescent Proteins
Source: Front Mol Biosci. 2021 Feb 3;8:633217. doi: 10.3389/fmolb.2021.633217 (PMC7983054; doi:10.3389/fmolb.2021.633217)
Supplement: Supplementary file 1 [file table1.docx]

**Supplementary Information.**

Figure S1. Graphical solution of eqs. (15) (blue lines) - and (20) (red lines) for Δ****** in RFPs. Directions of molecular coordinate axes are the same as in Fig. 7 of the main text. In XRFP and mPlum, the dashed curves correspond to a main absorption peak and the continuous curves correspond to a red-shifted (minor) absorption peak. Purple solid arrow is the solution of (15) and (20), falling into quadrant IV (see main text) and corresponding to the red shifted peak for XRFP and mPlum. Purple dashed arrows for XRFP and mPlum correspond to a main absorption peak and for eqFP670 corresponds to a solution found in quadrant III (see main text). Continuous dark green arrows correspond to a direction of ****** accepted in this paper. Dashed dark green arrow for XRFP corresponds to a main absorption peak. Light green arrow for mCherry depicts the direction of *** ***found experimentally in (Myskova et al., 2020). Purple dashed arrows correspond to the quantum mechanically calculated Δ****** vector in DsRed protein environment (List et al., 2012), described by different models, see text. Dashed straight lines depict the direction of oscillating transition dipole moment ******, also calculated in (List et al., 2012), for the chromophore in vacuum (light green) and in protein (dark green). Green continuous straight lines represent two possible directions of the ****** vector expected from our measurement of *γ* and selected direction of Δ******.

Figure S2A. Electrostatic potentials along the line connecting the centers of phenolate and imidazolinone rings calculated with MD simulations. The dots correspond to projections of positions of atoms CD2, CG2, CB2, CA2, and C2 (see Fig. 4 in main text) on this line. The straight line is a best linear fit. The electric field component *E_x_* is obtained as a negative slope of the line.

Figure S2B. Electrostatic potentials along the line perpendicular to *x*-axis and going through O2 atom, calculated with MD simulations. The dots correspond to projections of positions of atoms O2 C2, and CA2 on this line. The straight line is a best linear fit. The electric field component *E_y_* is obtained as a negative slope of the line.

Table S1. Components of the protein electric field obtained using eqs. (11) and (12) and from MD simulations. The fields were calculated for each Δ****** solution occupying one of the four quadrants on the *_x_*, *_y_* plane.

| Protein | Δ*** ***quadrant | I | II | III | IV | MD simulation |
| --- | --- | --- | --- | --- | --- | --- |
| *DsRed2* | *E_x_* (MV/cm) | 9.9 | 44 | 44 | **9.9** | *18* |
|  | *E_y_* (MV/cm) | 218 | 218 | 57 | **57** | *61* |
| *mPlum*  *(main)* | *E_x_* (MV/cm) | 19 | 36 | **36** | 19 | *36* |
|  | *E_y_* (MV/cm) | 231 | 231 | **44** | 44 | *88* |
| *mCherry pH8* | *E_x_* (MV/cm) | 18.3 | 36 | 36 | **18.3** | *15* |
|  | *E_y_* (MV/cm) | 233 | 233 | 42 | **42** | *40* |
| *mCherry pH11* | *E_x_* (MV/cm) | 12.9 | 41 | 41 | **12.9** | *8* |
|  | *E_y_* (MV/cm) | 219 | 219 | 57 | **57** | *68* |

Figure S3. Dependence of nonradiative decay rate (in logarithmic scale) on the change of electrostatic potential normalized to the shift of electronic density along *x*-direction, Δ*x*, at different values of parameter *η*. Theoretical fits for 5 proteins (black symbols), based on the Marcus theory are shown. mScarlet and eqFP670 data points (red squares) are not included in the fit (see main text).

Figure S4. Dependence of residual sum of squares for the best fits, similar to those shown in Fig. S3, as a function of parameter *η*.

Table S2. Variations of parameters Δx and Δy as a function of parameter .

|  | -0.2 | -0.16 | -0.12 | -0.1 | -0.09 | -0.08 | -0.016 |
| --- | --- | --- | --- | --- | --- | --- | --- |
| Δx, Å | 4.81 | 5.4 | 6.19 | 6.66 | 6.95 | 7.22 | 9.86 |
| Δy, Å | -0.96 | -0.86 | -0.74 | -0.67 | -0.62 | -0.58 | -0.16 |
| RSS | 0.0280 | 0.0149 | 0.00685 | 0.00589 | 0.00645 | 0.00784 | 0.0458 |

Table S3. Steric hindrances for rotation of phenolate around CB2 – CG2 bond. The structures were analyzed in Chimera and taken from pdb database (rcsb.org). The top row contains the number of amino acid residue (mCherry numbering) that come in close van der Waals with the phenolate part of chromophore, hinderin its rotation around CB2 – CG2 bond. No hydrogen atoms were included. In parentheses are shown the numbers of van der Waals contacts of a selected amino acid residue with any of CE1, CD1, CE2, CD2 atoms of the phenolate when the conformation chromophore is in planar conformation. The contacts that specifically hinder the rotation of phenolate are bolded. In red are the contacts that hinder clockwise rotation, defined by looking at the OH oxygen along the OH-CZ bond. Blue – contacts that hinder counterclockwise rotation.

| Protein | 63  (*α*-helix) | 161  (*β*-sheet) | 163  (*β*-sheet) | 197  (*β*-sheet) | 199  (*β*-sheet) | 214  (*β*-sheet) | 144  (*β*-sheet) | 70 |
| --- | --- | --- | --- | --- | --- | --- | --- | --- |
| mPlum  (Chain B)  2QLG pdb | Pro63  (**2**) | Met161  (1) | Met163  (**2**) | Ile197  (**6**=**4**+**2**) | Leu199  (4) | Glu215  (2) | Ser146  (2) | -- |
| mCherry  2H5Q pdb | Pro63  (**2**) | Met161  (1) | Met163  (**2**) | Ile197  (**6**=**4**+**2**) | Leu199  (3) | Glu215  (1) | Ser146  (2) | -- |
| DsRed2  1ZGO pdb | Pro63  (**1**) | Thr161  (1) | Lys163  (**2**) | Ala 197 | Leu199  (2) | Glu215  (**1**) | Ser146  (1) | Lys70  (**2**) |
| mScarlet  5LK4 pdb | Pro64  (**1**) | Ile162  (2) | Met164  (**2**) | Arg198  (**6**=**3**+**3**) | Leu200  (4) | -- | Ser147  (1) |  |
| eqFP670  4EDS pdb | Thr60  (**2**=**1**+**1**) |  | Met160  (2=**1**+1)) | Arg197  (**4**=**2**+**2**) | Leu199  (3) | Glu215  (**1**) | Asn143  (2) |  |

**SI References**

1. Myskova, J., Rybakova, O., Brynda, J., Khoroshyy, P., Bondar, A., & Lazar, J. (2020) Directionality of light absorption and emission in representative fluorescent proteins. *Proc. Nat. Acad. Sci.*, accepted.
2. List, N. H., Olsen, J. M. H., Jensen, H. J. A., Steindal, A. H., & Kongsted, J. (2012). Molecular-Level Insight into the Spectral Tuning Mechanism of the DsRed Chromophore. *Journal of Physical Chemistry Letters*, *3*(23), 3513–3521.
